# High-resolution temporal gravity field data products: Monthly mass grids and spherical harmonics from 1994 to 2021

**DOI:** 10.1038/s41597-023-02887-5

**Published:** 2024-01-13

**Authors:** Metehan Uz, Orhan Akyılmaz, C. K. Shum, Kazım Gökhan Atman, Sevda Olgun, Özge Güneş

**Affiliations:** 1 Dept. of Geomatics Eng., Istanbul Technical University, Istanbul, Turkey; 2 Division of Geodetic Science, School of Earth Sciences, The Ohio State University, Columbus, Ohio USA; 3 School of Mathematical Sciences, Queen Mary University of London, London, England; 4 Department of Physics, Ege University, Izmir, Turkey; 5 Dept. of Geomatics Eng., Kocaeli University, Kocaeli, Turkey; 6 Dept. of Geomatics Eng., Yıldız Technical University, Istanbul, Turkey

**Keywords:** Hydrology, Engineering

## Abstract

Since April 2002, Gravity Recovery and Climate Experiment (GRACE) and GRACE-FO (FollowOn) satellite gravimetry missions have provided precious data for monitoring mass variations within the hydrosphere, cryosphere, and oceans with unprecedented accuracy and resolution. However, the long-term products of mass variations prior to GRACE-era may allow for a better understanding of spatio-temporal changes in climate-induced geophysical phenomena, e.g., terrestrial water cycle, ice sheet and glacier mass balance, sea level change and ocean bottom pressure (OBP). Here, climate-driven mass anomalies are simulated globally at 1.0° × 1.0° spatial and monthly temporal resolutions from January 1994 to January 2021 using an in-house developed hybrid Deep Learning architecture considering GRACE/-FO mascon and SLR-inferred gravimetry, ECMWF Reanalysis-5 data, and normalized time tag information as training datasets. Internally, we consider mathematical metrics such as RMSE, NSE and comparisons to previous studies, and externally, we compare our simulations to GRACE-independent datasets such as El-Nino and La-Nina indexes, Global Mean Sea Level, Earth Orientation Parameters-derived low-degree spherical harmonic coefficients, and *in-situ* OBP measurements for validation.

## Background & Summary

GRACE (Gravity Recovery And Climate Experiment) satellites are designed to monitor spatiotemporal variations of the Earth’s gravitational field to improve our understanding of the changes in the global climate system, with the primary goal of properly mapping mass variations, including terrestrial water cycle, ice sheet and glacier mass balance, sea level change, and ocean bottom pressure variations. Data have been acquired for fifteen years, exceeding the anticipated five-year mission span from March 17 2002 through October 2017^1,2^. The collection of science mission data ended in October 2017 because of the age-related battery issue on GRACE-B in September 2017. GRACE-FO was launched in May 2018 as a successor mission to GRACE in order to ensure the mission’s continuity^3,4^. 11 consecutive months of data gap exist between GRACE and GRACE-FO missions. In addition, some of the monthly solutions are missing due to improper retracked orbit issues throughout the lifetime of satellites^5^. In recent years, there have been studies using different methods to fill this gap. While mostly focusing only on terrestrial water storage and excluding mass changes over oceans, few studies have also reconstructed long-term simulations of total water storage anomaly, i.e., the climate-induced mass anomaly, before the GRACE-era using different approaches and spaceborne data.

For instance, Humphrey and Gudmundsson^6^ simulated six different forms of mass anomaly from 1901 to 2019 using a statistical approach with three different land surface temperature (TEMP) and two different precipitation (PPT) data products as meteorological forcing datasets, and two different GRACE mascon solutions. Li *et al*.^7^, first separated both input (PPT, TEMP, Sea Surface Temperature (SST), and 17 other climate indices) and output (GRACE mascon mass anomaly) into spatial patterns and temporal modes using independent component /principal component analysis techniques. Then, the temporal modes were further decomposed using least squares and seasonal-trend decomposition to obtain trend, seasonal, inter-annual and residual components. Excluding the trend, each decomposed component is used in Artificial Neural Networks (ANN), AutoRegressive Exogenous (ARX) and Multiple Linear Regression (MLR) approaches independently to simulate/predict temporal modes of each component at grid cell scale. Finally, GRACE-estimated trend and spatial patterns are restored by adding them back to simulated modes. Thus, the long-term mass anomaly simulations from 1979 to 2020 are obtained. Differently from the two studies mentioned above, Löcher and Kusche^8^ calculated the monthly global gravity field by combining the low-degree gravity solution estimated from Satellite Laser Ranging (SLR) observations with the decomposed spatial patterns retrieved from the available monthly GRACE gravity field solutions using Empirical Orthogonal Functions (EOF). In this way, the hybrid monthly spherical harmonic gravity field models with GRACE-like spatial resolution, i.e., degree/order (d/o) 60 models from 1992 to 2019 are obtained though the solutions before 1994 are dominated by very large noise due to worse constellation of the SLR satellites prior to 1994.

In this study, we used an in-house developed hybrid deep learning architecture, namely Residual Deep Convolutional Autoencoder (ResDCAE), to simulate long-term high resolution (at monthly temporal and 1° × 1° spatial resolution) mass anomaly from 1994 to 2021. ResDCAE is based on the concept of residual learning and utilizes stacked autoencoders to increase learning efficiency and is developed considering the TensorFlow^9^ and Keras^10^ libraries. No prior detrending, deseasoning, or decomposing processes either to the input or to the output datasets are applied. Thus, the simulations avoid possible biasing or aliasing of long-term climate signals. In order to successfully simulate trend, interannual, and seasonal signals, we included both SLR-based coarse resolution mass anomaly and normalized Day of Year (nDOY) as additional input, where the latter is computed by dividing the DOY of the mid-day of that month by 365 (or 366). For this purpose, the monthly SLR-only spherical harmonic gravity field models (up to d/o 10)^8^ are used to effectively simulate the long-term trend, since the long-wavelength component of gravitational signals can be derived from SLR-only temporal gravity solutions. Interannual and seasonal signals, on the other hand, are simulated more accurately, thanks to nDOY. Because all geophysical signals are functions of time, using time epoch (nDOY) as an input acts as a constraint to obtain more realistic simulations. These novel ideas have already been tackled in recent study by Uz *et al*.^11^ comprehensively, but Swarm-derived mass anomaly instead of SLR mass anomaly is used to obtain the long-wavelength component of gravitational signals between January 2014 and January 2021. Here, we focused on longer-term simulation considering a similar strategy and provided global simulations including both continents and oceans. The simulated mass anomalies are validated using the internal and external validation data. Furthermore, each monthly mass anomaly simulations are also converted to global geopotential field models expressed in spherical harmonics complete to degree and order 200.

## Methods

### Residual deep convolutional autoencoders

Convolutional Neural Networks (CNNs) are special types of neural networks and are useful for processing data with a grid-like architecture, such as images or time series data^12,13^. In particular, CNNs utilize the convolutional layers, which are linear operators and convolve the input with the set of filters. Therefore, the CNNs can be considered as spatial feature extractors by their layered structure. For this reason, CNNs have been widely used for problems such as filling the data gap in remote sensing^14^, land surface temperature reconstruction^15^, etc. In this manner, the relationship between the output vectors **a** of the consecutive layers of CNNs is represented as:

1 $${a}^{(l+1)}=\sigma ({a}^{(l)}\ast {W}^{(l)}+{b}^{(l)})$$

where ∗ denotes convolution operator, σ(·) is the activation function, with weight matrix **W** and bias vector **b** while the superscript indicates the layer ID. In addition, CNNs mainly comprise three types of layers: convolutional layer, pooling layer, and fully connected layer. Deep Convolutional AutoEncoder (DCAE) is a deep learning architecture that may be considered as a combination of two neural networks, namely encoder and decoder^16–18^. In particular, the encoder maps the input space into a lower-dimensional latent space by h = f (**x**), while the decoder maps the latent space into the reconstruction space by r = g (h) and here x is the input vector. By this way, the network learns the representations of input data by reducing the dimensionality of data in either a supervised or an unsupervised manner. Basically, the high-abstraction features are learned while mapping through an internal representation, or code, h, in the intermediate layer. In addition, the distinction between standard AutoEncoder (AE) and DCAE is the utilization of convolutional layers. Accordingly, DCAE takes input data and maps it to h,

2 $$h=\sigma \left(Wx+b\right)$$

where σ denotes the activation function, **W** and **b** are the weight matrix and bias vector of the encoder. Accordingly, the output of the decoder is given as follows:

3 $$r=\sigma \left(\widehat{W}h+\widehat{b}\right)$$

One of the common themes in deep learning architectures is that the deeper the network, the more advantageous and the better the modelling performance. However, there is the problem of vanishing/exploding gradients due to the successive calculation of gradients with respect to the gradient from the previous layer. To overcome this problem, a residual neural network was proposed by He *et al*.^19^, which led to an effective strategy for developing deeper neural networks. The main reason for this is the fact that instead of calculating gradients over F(x) which represents the mapping, gradients are calculated over F(x)+x, by introducing the skip connections between layers. Accordingly, output y is obtained by the combination of the input and output of the earlier layer as follows.

4 $$y=F\left(x,{W}_{i}\right)+x$$

where *F*(*x,W*_*i*_) is the residual mapping and *W*_*i*_ corresponds the i-th weight matrix in the hidden layer weighted value of the layer. It should also be noted that the dimensions of the x and y must be equal. The residual learning strategy is successfully applied to problems such as classification^20^, the spatiotemporal estimation of citywide crowd flows^21^ and influenza trends^22^.

The proposed architecture is given in Supplementary Fig. S1 and based on the combination of DCAEs and CNNs with the concept of residual learning. The main reason for this implementation is that it improves learning efficiency by developing deeper structures with the help of residual learning. Accordingly, the structure of the proposed network may be divided into three parts as follows:

1. Convolutional building block: The developed architecture is based on the use of the CNN which consists of two convolutional layers with 126 and 63 filters in each layer, respectively, followed by a dense layer with 21 neurons. It should also be emphasized that the size of the filters and neurons is chosen according to the channel size of the input. In addition, for each convolutional and dense layer, an Exponential Linear Unit (ELU) activation function is used throughout the network, and each convolutional layer also has a regularizer to prevent overfitting. Besides, a single convolution layer with 21 filter sizes is utilized as the residual connection. In the stage of selecting hyperparameters of CNN, we have considered the lowest generalization error subject to runtime and memory constraints. In addition, elastic net is utilized as a regularization method which combines the lasso and ridge regulators^23^, with penalty value of 10^−4^. It is also worth noting that the reason for adopting the ELU as an activation function is that it allows to negative outputs which leads to adjusting weights and biases in the correct direction during the iterative optimization process.
2. DCAE building block: The structure of the DCAE model consists of five convolutional layers with a gradually increasing filter size from 21 to 316. Each convolutional layer is followed by a maxpooling layer with a pool size 2 × 2 and 3 × 3 respectively. In the intermediate layer, which is also known as latent space, a flattened layer and a fully connected layer with 400 neurons are used. In this manner, in the decoding part, the fully connected layer of 400 neurons is followed by the four transposed convolutional layers, symmetric to the encoder part. In addition, the ELU activation function is used in each layer of the network.
3. Regression building block: To complete the end-to-end image-to-image regression task, a CNN-based structure is employed, which consists of two convolutional layers with 21 and 14 filters, respectively, followed by a dense layer with 1 neuron. Furthermore, the ELU activation function is applied to each convolutional layer except the dense layer.

Accordingly, the input features first pass through the DCAE building block and are concatenated to the output features of this block. Further, this serves as the input features to the convolutional building block, and before the concatenation, input features are convolved. In order to complete image-to-image regression, regression building blocks are employed as the final layers of the model. Therefore, the model has 6 consecutive DCAE and convolutional blocks and 1 regression block. Regarding the implementation of the network, the Adamax optimizer is utilized with the initial learning rate equal to 10^−3^ and a batch size of 27. In addition, the learning rate is reduced by a factor of 0.8 when learning stagnates by monitoring the validation loss. Furthermore, early-stopping is implemented to mitigate overwriting. According to this, validation loss is selected as the monitored metric, and the training procedure is stopped if no improvement is seen for 25 epochs. The Huber function has been selected as the training loss function since it is robust against outliers and has fast convergence to near negligible loss. The main reason for this implementation is that the input consists of various earth observations that have different characteristics. The training is performed on a single NVIDIA Tesla P100-PCIE-16GB GPU. It should also be noted that the full memory capacity of the graphic card is used, and the running time of the model is about 45 minutes.

### Mitigating trend error in backwards extrapolation

Our objective is to provide monthly gravity field data products similar to those within the GRACE era, even for the pre-GRACE period. Achieving this goal involves extrapolating data backwards in time, which is a complex task. Extrapolation should be performed with caution, especially when dealing with non-stationary processes, as is the case in our study. Non-stationarity in Earth and environmental systems, such as spatiotemporal changes in Earth’s water mass, primarily results from the inherent secular trend signal. This signal alters the mean rather than the signal variance and may or may not follow a linear pattern. Factors like climate change, human interventions, and low-frequency internal variability, such as the Atlantic multidecadal oscillation, affected by the slow dynamics of ice sheets and the ocean, contribute to this non-stationarity^24^. This introduces the challenge such that the behaviour of the signal outside the training data period (in our case, the GRACE and GRACE-FO period) may differ from that within the training data time span, even if the seasonal amplitudes remain relatively consistent^25^. Like all data-driven approaches, deep learning (DL)-based methods adjust their parameters through optimization algorithms to find the best fit to available output data based on the corresponding input data. This optimization aims to establish a statistically optimal mapping from input to output, limited to the training data period. Consequently, deep learning models typically perform well when producing or predicting outputs for new input values falling within the range of input data used for training. However, predicting outcomes outside the range of the training data, referred to as ‘out of sample prediction,’ can be challenging. In other words, a deep learning model can provide reasonable results within the hyperspace defined by the boundary of the training data set, which can be seen as a high-dimensional interpolation, as long as the number of input data variables is fewer than 100^26^.

In the case of mass anomaly, an efficient extrapolation requires a priori knowledge about the signal behaviour in the extrapolation regime, i.e., in the time span out of the training data period. Unfortunately, this kind of information is usually not available, at least globally and at grid cell scale. However, the main differences of the mass anomaly signal as well as of the input climate data signal in the extrapolation regime from those in the interpolation regime (i.e., training data span) are in the long-term trends while the seasonal amplitudes do not vary much (see e.g., Supplementary Fig. S2). Therefore, the main errors of extrapolation are due to mismodeling of the trend component which is retrieved from the training data. Some studies^6,7^ estimate and remove a linear trend using the data in the GRACE and GRACE-FO mission spans before calibrating their models based on residual signal and then extend and restore this trend to the extrapolation regime by assuming that their estimated linear trends also hold out of the training data period. Such an assumption is too optimistic and may not be valid globally. A typical example is the surface mass balance estimates at polar regions, e.g., Greenland and Antarctica (see The IMBIE team^27,28^) exhibit relatively lower mass change rates until late 1990s followed with an onset of dramatic increase in mass loss after 2000 due to the accelerated ocean-driven melting of the ice sheets. Similar extrapolation errors are also reported^29^ for the long-term static gravity field solutions with co-estimated (TVC) time-variable coefficients (secular and seasonal periodic components), e.g. GOCO06S^30^, by evaluating the differences of mass anomaly from the monthly GRACE-FO solutions and those extrapolated from the static field with TVC computed from the data solely within the GRACE era, suggesting that the static gravity models with TVC cannot be used for long-term (>2-3 years) extrapolation and at least should be frequently updated with the newly available GRACE-FO data, e.g. for mass change studies as well as for improved precise orbit determination of low earth orbiting satellites. Also, Mouginot *et al*.^31^ and Rignot *et al*.^32^ discuss these mass changes for the last 40 years. Mouginot *et al*.^31^ reported variability in the mass balance of the Greenland Ice Sheet since the 1980s, along with a sixfold increase in mass loss. This has resulted in a significant 13.7 mm contribution to global sea level rise since 1972, with half of this effect occurring during the period from 2010 to 2018. According to Rignot *et al*.^32^, the primary cause of mass loss in Antarctica is the glacier flow near warm, saline circumpolar deep-water regions, particularly in East Antarctica, with significant implications for future sea-level rise. In addition to these, Caceres *et al*.^33^ studied the land water storage except for the glaciers mass change. They reveal that from 1948 to 2016, continents contributed to a sea-level rise of 34–41 mm, with glacier mass loss responsible for 81% of the cumulative loss and land water storage anomalies accounting for the remaining 19%. Climate-driven land water storage anomalies are notably influenced by precipitation and linked to El Niño Southern Oscillation, although uncertainties persist in modelling these anomalies, particularly in relation to irrigation water use and artificial reservoirs.

The power of DL, besides the computational resources, is attributed to the number of training data, that is, higher the number of data higher the accuracy can be achieved by large neural networks whose parameters are updated through deep learning algorithms (Aggarwal^34^, pp. 3).

After training the deep learning model with initial training and test data (see section Data Architecture for details of the training and test data) within the GRACE/-FO era and simulating the a priori monthly mass anomaly of 3-years backwards in time (i.e. from April 1999 to March 2002), here we applied a step-by-step piecewise trend correction approach where at each step the number of training data is incrementally increased backwards in time. The overall approach can be summarized as follows. We start with retraining our deep neural network after removing the first three years of the initial training data within the GRACE/-FO era (i.e., 3 years of data starting from April 2002 to March 2005) without altering the network architecture or any of the hyperparameters or the learning algorithm. The retrained model was then used to simulate global 1° × 1° monthly mass anomaly grids for the period coinciding with that of the removed 3-years of initial training data. For each grid cell, the linear trends from both the simulated and the corresponding original monthly mass anomaly data in the initial training set in these 3-years were estimated by least squares fit, and the difference between the two trends was computed. The computed trend difference at each grid cell was then applied to correct the simulated mass anomaly of the earlier 3-years (i.e., the monthly mass anomaly from April 1999 to March 2002). These new corrected simulations were added to the initial training data set which now constitutes the extended training data set for the next iteration. With the extended training data, the procedure above was repeated with removing the first 3-years (i.e., this time April 1999 to March 2002) and computing trend-corrected mass anomaly simulations for the previous 3-years (i.e., April 1996 to March 1999). This step-by-step correction process was applied once again with the updated training data from previous iteration so that the trend-corrected mass anomaly simulations from January 1994 to the beginning of GRACE/-FO era (i.e., April 2002) were completely obtained. The efficiency of the above procedure and the adequacy of the chosen step-size, i.e., 3-years have been verified with results shown in Technical Validation section.

### Descriptions of the input and output data of our DL-based simulation model

Our DL architecture possess the multichannel input consisting of seven variables and a single output variable. Input are monthly coarse resolution SLR-only mass anomaly and five different Hydroclimatic/meteorological Variables (HV) from ERA5 (European Centre for Medium-Range Weather Forecast-ECMWF Reanalysis-5) as well as normalized (Day-of-Year) time epoch of these monthly dataset, i.e., nDOY. The five HV from ERA5 are PPT, TEMP, SST, Cumulative Water Storages Changes (CWSC) and mass anomaly retrieved from ERA5 model data while the single output is the monthly CSR RL06 Mascon (CSRM) mass anomaly solutions. The details of both input and output are given in the following.

### GRACE mass anomaly data

The monthly mass anomaly of GRACE/-FO is derived from CSR RL06 Mascon (CSRM) solutions^35,36^. The time span of this dataset is fragmented into two main parts that are April 2002–June 2017 for GRACE and May 2018–to 2021 for GRACE-FO missions. There is an 11 successive months of so-called intermission data gap between GRACE and GRACE-FO, but there are also missing months during the operational time period of each mission. All the standard post-processing corrections have been applied to CSRM models, i.e., degree 1 correction^37^, replacement of C_20_ and C_30_ coefficients^38^, Glacial Isostatic Adjustment (GIA)^39^ and Ellipsoidal correction^40^. The monthly mass anomalies are calculated considering the mean baseline between 2004.0 and 2009.9999. Besides, while the temporal resolution is monthly for CSRM, the spatial coverage is global, with a spatial sampling resolution of 0.25° × 0.25°. We resampled the original CSRM to 1.0° × 1.0° grids considering the native resolution of CSRM which determines the spatial resolution of our target mass anomaly simulations.

### SLR mass anomaly data

The monthly SLR-only spherical harmonic gravity field models^8^ up to d/o 10 are provided by Dr. Anno Löcher from the Astronomical, Physical, and Mathematical Geodesy Institute of Bonn University via personal communication. The coarse resolution SLR-only monthly gravity field solutions are available from November 1992 to January 2021. The monthly mass anomalies from SLR-only models are calculated by applying post-processing to the spherical harmonic coefficients of those models after removing the same mean baseline (2004.0–2009.9999) with CSRM for consistency. A 1500 km Gaussian smoothing filter is applied, i.e., the filter radius is decided after applying both 1000 and 2000 km, but the optimum results are obtained from using the 1500 km radius showing a compromise between signal loss and noise reduction. No de-striping filter was applied as suggested by the data provider (Dr. Anno Löcher, pers. comm.) Mass anomalies are directly calculated for each 1.0° × 1.0° grids on the globe including both ocean and land.

### ERA5 data

The ERA5 dataset is released by the European Centre for Medium-Range Weather Forecast (ECMWF - https://cds.climate.copernicus.eu/) and consists of both monthly averaged and hourly sub datasets. Besides, ERA5 has two different parameter levels, i.e., single and pressure levels. We used the monthly averaged single level from 1979 to present;^41^ a subset of ERA5 considering the timespan of the available SLR-only input data. Therefore, ERA5 was downloaded and used from November 1992 to January 2021. The basic input HV data are chosen in order to be used in the DL algorithm which are PPT, TEMP, SST, RunOff (RO), evapotranspiration (ET), snow water storage (SnWS), soil moisture storage (SMS) and Canopy Water Storage (CnWS). The input ERA5 mass anomaly and cumulative water storage changes (CWSC) are calculated using these downloaded variables applying the equations given in e.g., Uz *et al*.^11^, and Mo *et al*.^42^. Similar to the CSRM and SLR-only mass anomaly, each input data from ERA5 is referenced to the mean baseline, i.e., 2004.0–2009.9999, by removing its mean within this baseline period. Finally, all HV data are resampled from 0.25° × 0.25° to 1.0° × 1.0° to ensure consistency between all input and output data.

### Data architecture

There are two considerations while designing the DL architecture which are addressed with the questions; (i) how do the temporal patterns of input and output data for each grid vary throughout their own time-spans? and (ii) how do the temporal correlations change with respect to time lags? The answers to these questions could give information about how many additional layers should be considered for the input data variables. In other words, how many channels should be used for the input data. We answered these questions by inspecting the Partial AutoCorrelations (PAC) computed for varying time lags in Amazon River basin. The Amazon River basin was chosen because it is a good example of reserving climate change signatures^43^. The grid closest to the centre of the basin, according to the basin boundaries from Total Runoff Integrating Pathway (TRIP) database^44^, was selected, and the PACs for each input and output were computed and plot. The time series of the input and output data and their corresponding PACs are given in Supplementary Fig. S2 and, except for SLR-only mass anomaly, all other input and output data show more or less a certain annual signal pattern. The SLR-only mass anomaly between December 1992 and December 1993 has higher variations with respect to those during the rest of the time span. Similar results are also reported in Löcher and Kusche^8^. The obvious reason for this is that the SLR-only models in the first couple years were computed without using the Stella satellite, which joined the constellation in September 1993 and is an important part of SLR-only temporal gravity field recovery due to its low polar orbit and hence providing more redundant sets of normal equations for gravity inversion. Thus, the time series of SLR-only mass anomaly includes even higher noise until September 1993 (Dr. Anna Löcher, pers. comm.). On the other hand, PACs are calculated from the time series of input and output, starting from zero lag up to a 12-month time lags. All correlations are computed throughout the GRACE/-FO time period and are illustrated in Supplementary Fig. S2e. It is clearly seen that almost all correlations reduce to below zero at a two-month lag and are almost zero beyond the two-months. Thus, we decided to set the number of additional layers to 2, i.e., the successive two months of relevant time epoch of input, i.e., *t* and *t-1*.

According to the pre-analyses above, DL architecture is constituted considering both the time-span of SLR-only models with lower noise level and the computed temporal correlations. In addition, our initial training and testing data sets are randomly selected from the GRACE/-FO time period. While the total number of initial training months is 135, the number of testing months which is kept unchanged when applying the trend correction procedure is 57. The entire time series of predicted/simulated monthly mass anomaly cover both the pre-GRACE era as well as the existing data gaps within the GRACE/-FO time period after the step-by-step trend correction was applied. The number of predictions where no GRACE/-FO data is available is 136, starting from January 1994 due to a higher noise issue in SLR-only solutions between 1992 and 1994.

## Data Records

The simulated monthly dataset is released both in the form of gridded mass anomalies with and spherical harmonic coefficients in accordance with the ICGEM (International Center for Global Earth Models) format. These datasets are available in figshare repository^45^. The datasets cover the time span from January 1994 to December 2021, hence provides simulations for 324 months in total. The gridded dataset is available to users with data format identical to official CSR mascon data products in figshare as netcdf file with four variables: lat, lon, time and mass anomaly. Lat and lon are latitude and longitude vectors of dimension 180 × 1 and 360 × 1, representing the positions of the centre of 1.0° × 1.0° grid cells on the surface of the Earth. The mass anomaly represents the relative change in the water mass with respect to the mean baseline (2004.0-2009.9999) in terms of cm Equivalent Water Height (EWH); thus, the associated netcdf file has a dimension of 324 × 180 × 360 while the time is a column vector of dimension 324 × 1 with days since 1994 01 01 00 00 00.0 UTC. The monthly mass anomaly in the form of spherical harmonic coefficients from degree 2 up to degree/order 200 were released as ASCII files. The file naming convention similar to the official GRACE data processing centers was adopted, i.e., determined by the year and the day of the year corresponding to the first and last day of the respective month. Each file has a header that contains information regarding constant values.

## Technical Validation

This section evaluates the simulation performance of our models and summarizes the findings in two categories: internal and external validation. Internal validation is performed by comparing our simulations to CSRM mass anomaly solutions and to those from previous studies in terms of common mathematical goodness-of-fit metrics such as Root Mean Square Error (RMSE), Nash - Sutcliffe efficiency (NSE^46^), and Pearson Correlation Coefficient (PCC). On the contrary to internal validation, the simulated mass anomalies (or spherical harmonic coefficients derived from them) are validated externally by comparisons to GRACE-independent datasets, such as the long-term surface mass balance estimates of Greenland, the El Niño/La Niña SST index, global barystatic mean sea level changes, degree 2 order 1 spherical harmonic coefficients (a.k.a. C_21_, S_21_) retrieved from daily Earth Orientation Parameters (EOP) series, degree 2 order zero spherical harmonic coefficient (i.e., C_20_) from SLR and *in situ* Ocean Bottom Pressure observations.

### Internal validation

#### Comparison of different input scenarios

The first step in our internal validation process is to assess the GRACE-like mass anomaly simulations generated by our deep learning algorithm, namely the ResDCAE model. Our objective is to determine how the inclusion of SLR and nDOY inputs in the model affects these simulations. To do so, we have devised four distinct simulation scenarios, which we refer to as DL models. These scenarios are grouped based on whether they incorporate SLR and/or nDOY inputs, while all DL models consistently include all four ERA5 layers.

To make it easier to distinguish between these various combinations, we have assigned specific names to them: Sol1 (which includes both SLR and nDOY), Sol2 (which includes only SLR), Sol3 (which includes only nDOY), and Sol4 (which excludes both SLR and nDOY). We then compare the mass anomaly simulations from these four solutions with the reference mass anomaly data from original CSR Mascon solutions. In each of these comparisons, we calculate two commonly used metrics, namely RMSE (Root Mean Square Error) and NSE (Nash-Sutcliffe Efficiency), for all test months. These metrics allow us to effectively assess the performance of the models and gain insights into the impact of different input configurations.

**Fig. 1:**
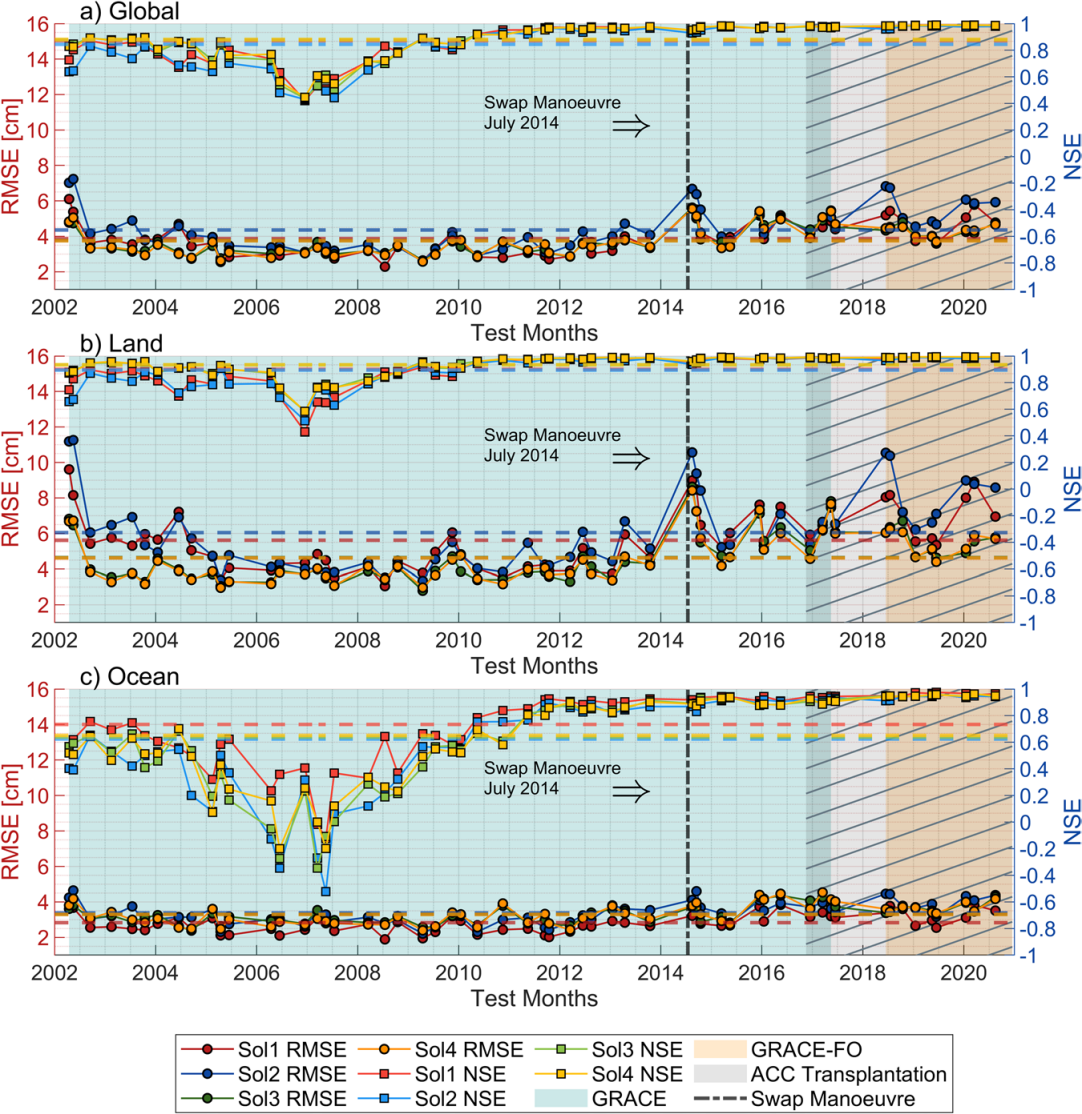
Comparison of overall RMSE and NSE values computed over (**a**) land + ocean (global), (**b**) land-only and (**c**) ocean-only areas on Earth from 57 test months.

From April 2002 to August 2020, RMSE and NSE metrics for each of the randomly chosen 57 test months and their overall mean are computed and shown in Fig. 1. The overall mean values of the metrics are also listed in Table 1. Furthermore, the metrics are separately computed over (i) entire globe (land + ocean), (ii) land-only, and (iii) ocean-only areas. All three scenarios (i-iii) exhibit a level of accuracy in retrieving the missing test months, with RMSE of 4 cm, 5 cm, and 3 cm, respectively. The corresponding NSE metrics are computed as 0.86, 0.91, and 0.68. A similar comparison was made by Uz *et al*.^11^ between April 2014 and September 2020 for the thirteen test months but only over land areas. The simulative performance of test months that either encompass the ACC (accelerometer) transplanted^47^ time period or those which uses the piled data from two successive months to solve for the corresponding CSRM mass anomaly is worse than those within the other time spans (see Fig. 1 of Uz *et al*.^11^). The same is also reported over oceans (see Fig. 2 of Chen *et al*.^48^). From this point of view, while the CSRM products of GRACE after November 2016 are calculated using transplanted ACC data to mitigate the effect of the GRACE-B battery issue^49^, the ACC transplantation has been carried out since the start of the GRACE-FO mission because the standard ACC data derivation procedure from Level-1A (L1A) to Level-1B (L1B) does not ensure sufficient accuracy for gravity field recovery^50,51^. Thus, the Science Data System (SDS) produces and distributes the transplanted and calibrated ACC data product on a regular basis. Additionally, the specifics of Level-2 (L2) data products, metadata including whether the ACC transplantation is applied or piled data from consecutive months used for gravity inversion, are listed in Table 2 of the SDS Newsletter(https://isdc.gfz-potsdam.de/grace-isdc/grace-gravity-data-and-documentation/). As expected, the RMSE of all solutions over land are greater than those over the oceans. This is because the mass anomaly signal over land is stronger while those over ocean has lower magnitude which is dominated by noise. Therefore, the computed NSE over ocean are lower due to the low signal-to-noise ratio of CSRM over ocean at grid cell scale.

**Table 1:** Overall monthly RMSE (cm) and NSE metrics for 57 test months.

	Region	Sol1	Sol2	Sol3	Sol4
**RMSE**	**Global - RMSE**	3.9	4.4	3.8	3.7
	**Land - RMSE**	5.6	6.0	4.6	4.6
	**Ocean - RMSE**	2.8	3.4	3.3	3.3
**NSE**	**Global - NSE**	0.87	0.85	0.88	0.88
	**Land - NSE**	0.90	0.89	0.93	0.93
	**Ocean – NSE**	0.73	0.62	0.64	0.65

**Fig. 2:**
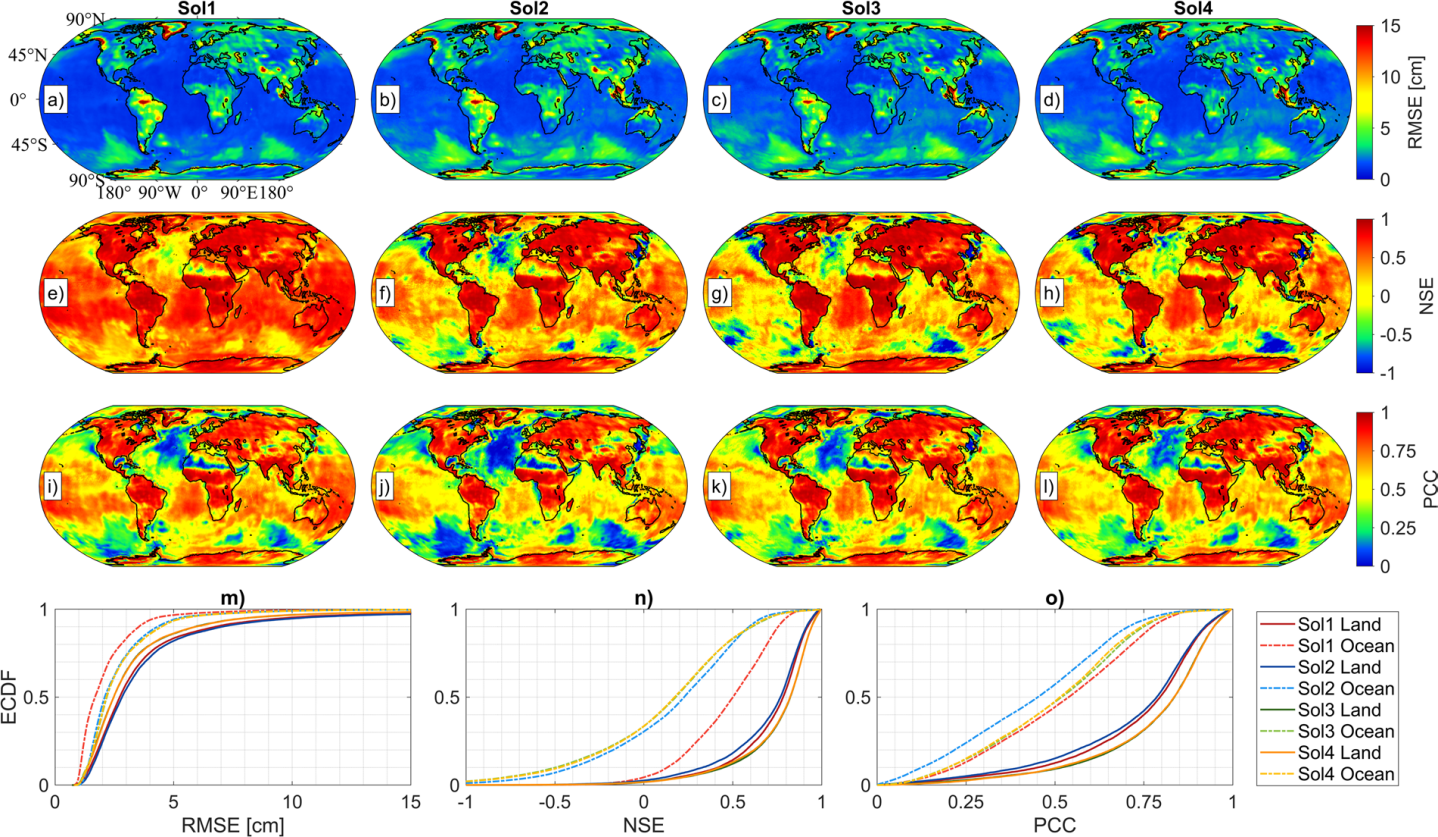
Spatial distributions of RMSE (**a**–**d**), NSE (**e**–**h**) and PCC (**i**–**l**) metrics of different solutions (Sol1, Sol2, Sol3 and Sol4) calculated from 57 test months and the ECDF illustrations of (**m**) RMSE, (**n**) NSE and (**o**) PCC for all solutions over land and over oceans on Earth.

**Table 2:** Correlations between ResDCAE-, CSRM-, EOP- and SLR-derived degree-2 spherical harmonic coefficients ∆C_20_, ∆C_21_ and ∆S_21_ within GRACE/-FO and pre-GRACE era.

		Pre-GRACE	GRACE/-FO
Δ*C*_20_	ResDCAE - SLR	0.70	0.80
	ResDCAE - CSRM	—	0.96
	CSRM - SLR	—	0.80
Δ*C*_21_	ResDCAE - EOP	0.58	0.65
	ResDCAE - CSRM	—	0.94
	CSRM - EOP	—	0.68
Δ*S*_21_	ResDCAE - EOP	0.65	0.85
	ResDCAE - CSRM	—	0.96
	CSRM - EOP	—	0.86

(Note that the pre-GRACE era is not shown for comparisons to CSRM because there are no CSRM solutions available for that period.).

The lowest RMSE are found in time series between 2004 and 2010, which is attributed to the better orbit configuration and availability of telemetry data with minor gaps during this time span (Fig. 1). Additionally, the NSE over oceans is at its lowest level within this time period while the RMSE is still minimum (~2 cm), implying that the DL algorithm successfully mitigated the high frequency spatiotemporal ocean mass change errors of CSRM at grid cell scale (Fig. 1c). There is a significant jump in RMSE of all simulations, which is clearly seen in the comparison over land-only areas (Fig. 1b), around August 2014. According to the August 2014 SDS Newsletter, the swap manoeuvre, during when the satellite twins exchange positions^52,53^, was carried out in July 2014. It may have impacted the satellite observations in August 2014 and in the following few months.

The overall metrics in Table 1 reveal the following. Over land, the RMSE and NSE of Sol1 and Sol2 are higher and lower, respectively, than those of Sol3 and Sol4. However, the metrics of Sol3 and Sol4 are nearly identical. The primary distinction between Sol1-2 and Sol3-4 is the presence of SLR-only mass anomaly as a training input. It is expected that SLR-only mass anomaly would be noisier and will propagate to the Sol1 and Sol2 simulated models. Thus, mass anomalies that incorporate the spatiotemporal variations of the SLR-only mass anomaly through input are noisier than those that do not. On the other hand, the relationships of the metrics with the simulation models over oceans are different from those over land. While Sol2 metrics continue to have the greatest RMSE and the lowest NSE values over the ocean, Sol1 metrics reveal the opposite. Differently from Sol2, Sol1 uses normalized time (nDOY) as an additional training input. Although Sol1 exhibits propagated noise from input SLR-only mass anomaly, the simulated model is also associated with the temporal changes of nDOY input. On Earth, the oceanic regions have more complicated dynamics, and gravitational signals are influenced by the higher-frequency mass redistributions at oceans. Thus, the temporal variations retrieved by the aid of nDOY parameter may guarantee to provide more accurate description of the signal throughout the ocean. The metrics computed considering both land and ocean (global) grids are used to assess which simulation model has the best mathematical fit.

The simulation models are further evaluated based on the spatial distribution of RMSE, NSE, and PCC by the illustration given in Fig. 2. These metrics are calculated for each 1.0° × 1.0° grid cell from monthly differences between DL-based simulations and corresponding CSRM mass anomaly in the 57 testing months. While the rows of Fig. 2 correspond to RMSE (a, b, c, and d), NSE (d, e, f, and g), and PCC (h, i, j, and k), the columns correspond to simulations Sol1 to Sol4, from left to right. The highest RMSE is seen in the same regions for all simulations, i.e., the Amazon, Ganges, Greenland, and Gulf of Alaska. This result is also observed by previous studies (e.g., see Fig. 2a of Humphrey and Gudmundsson^6^, Fig. 2d of Li *et al*.^7^, Fig. 4j-1 of Mo *et al*.^42^ and Fig. 3 of Uz *et al*.^11^). These basins are hydrologically active in terms of signal variations and are the main contributors to ice sheet melting areas on Earth. For example, the Amazon is the largest drainage basin and is subject to the largest seasonal changes that can be surpassed by variations as much as 1 m of EWH in Total Water Storage (TWS) globally^54^. The Ganges basin is under the coupled effects of groundwater depletion due to human intervention for irrigation^55^ and the melting of ice sheets in High Mountain Asia glaciers. The ice sheet mass loss in Greenland and the Gulf of Alaska, moreover, contributes to global sea-level rise^27,56^. The mass anomaly signals and variations in these regions are higher than those in other basins on Earth. Thus, the discrepancy between CSRM and simulated mass anomaly is sourced from this outcome, and systematically worse or higher RMSE are calculated at these regions. In addition, the spatial distribution of RMSE for Sol1 and Sol2 is more intense than for Sol3 and Sol4 due to the propagation of the noise of the SLR-only input into the simulations. The Empirical Cumulative Distribution Functions (ECDF) of RMSE over land are illustrated in Fig. 2m. While 80% of RMSE are below 5 cm for all simulations, there is a significant difference between SLR-only mass anomaly included simulations, i.e., Sol1, Sol2, and not included ones, i.e., Sol3, Sol4. Similarly, NSE and PCC values over land can be evaluated by considering the spatial distributions of these metrics as shown in Fig. 2. Both NSE and PCC of all simulations are almost zero throughout arid regions on Earth, e.g., North Africa. These results are similar to those of Uz *et al*.^11^. Although the RMSE at arid regions are almost the same and having the lowest values, there is also very little correlation between CSRM and simulated mass anomaly which can be explained by the low signal-to-noise ratio of CSRM at these regions.

**Fig. 3:**
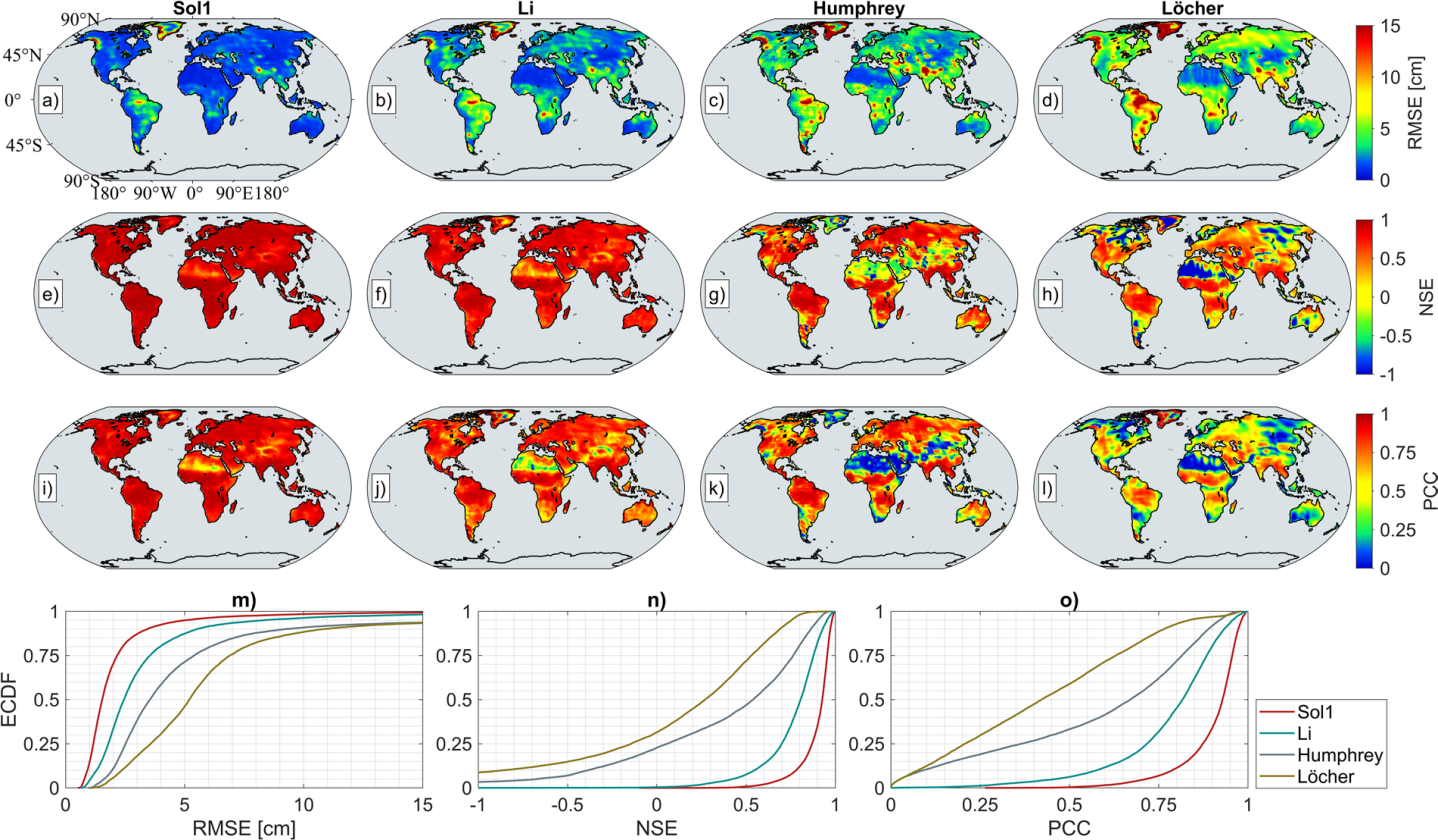
Spatial distributions of RMSE, NSE, and PCC computed over land areas (excluding Antarctica), with each measure represented in separate lines. Specifically, (**a,e,i**) correspond to Sol1, (**b,f,j**) to Li *et al*.^7^, (**c,g,k**) to Humphrey and Gudmundsson^6^, and (**d,h,l**) to Löcher and Kusche^8^. These calculations are based on 175 common months of mass anomaly solutions from all four studies. Additionally, the figure includes Empirical Cumulative Distribution Function (ECDF) plots for (**m**) RMSE, (**n**) NSE, and (**o**) PCC.

The simulative performances of the DL models over oceans are also demonstrated spatially with the same metrics. The geophysical dynamics are more complex in oceans. This complexity is sourced from the oceanographic variables and their temporal variations. Thus, the variations of these variables are more difficult to model when compared to land and seem to degrade the simulation performances over oceans in all four simulations. As shown in Fig. 2a–d, the RMSE at high latitude regions are even higher compared to the other parts. These regions may be influenced more by polar climatic characteristics. On the other hand, NSE and PCC over the ocean possess the lowest values not only in areas that are close to polar regions but also in different parts of the ocean, i.e., the Atlantic Ocean. There is a significant difference between Sol1 and other simulations, based on the ECDF of RMSE and NSE scores computed over the oceans (see Fig. 2m,n and Table 1). The simulations of Sol1 clearly exceeds other simulations in terms of metrics meaning that the oceans are better modelled using the input combination adopted for Sol1, i.e., when both SLR-only mass anomaly and nDOY are included as additional input data. For example, among all only the NSE of Sol1 is above zero, which indicates that only Sol1 modelled the mass change over oceans realistically. In general, if the NSE of a simulation model is below zero it means that the mean of observation is better than the simulation results^57^. According to our analyses so far, Sol1 and Sol2 suffer from propagation of noise in SLR-only input mass anomaly over land, but Sol1 provides better simulations over oceans.

#### Comparison with previous studies

Based on the comparisons among the four DL simulation models in the previous section we pick the simulation results of Sol1 as our final reconstructed data products. Thus, the simulations of Sol1 are compared to the previous similar studies of Humphrey and Gudmundsson^6^, Li *et al*.^7^, and Löcher and Kusche^8^ (which are called Humphrey, Li, and Löcher in the rest of the paper, respectively) regarding the performance metrics. The chosen reconstruction of Humphrey uses both JPL RL06 mascon and ERA5 HV, and the spatial resolution is 0.5° × 0.5° covering the time span from January 1979 to July 2019. The simulated mass anomaly of Li is calculated based on the CSR RL06 mascon with a spatial resolution of 0.5° × 0.5° and covers the time span between July 1979 and June 2020. On the other hand, hybrid models of Löcher were released as spherical harmonic coefficients up to degree and order 60 from November 1992 to January 2021. Thus, mass anomaly is calculated from the model coefficients with spatial resolution of 1.0° × 1.0°, removing the mean-field between 2004.0 and 2009.9999. To ensure consistency, Humphrey and Li’s mass anomalies are also calculated by removing this mean-field and up-sampled to 1.0° × 1.0° grids. The time period of comparison of all studies was chosen to be within the GRACE/-FO time period. In total, comparisons based on overlapping 175 months are made, and the illustration of the metrics is given in Fig. 3. The columns of Fig. 3 are represented by Sol1, Li, Humphrey, and Löcher from left to right, respectively. Similar to the results of the four DL simulations as shown in the previous section, the RMSE of all models are higher in hydrologically dominant regions on Earth. The order of RMSE performances of the models from best to worst with respect to CSRM mass anomaly are Sol1, Li, Humphrey, and Löcher. A similar performance order is also clearly seen for NSE and PCC. The illustrated ECDF in Fig. 3m,n,o also represent the model accuracies prominently. Throughout the GRACE/-FO era, the Sol1 simulation has the lowest spatial RMSE and the highest NSE and PCC with CSRM. In contrast to our DL-based simulation, the Li, Humphrey, and Löcher used different approaches or estimation strategies, therefore the long-term trend of each study may be different from the other. For consistency, the RMSE, NSE and PCC metrics are recalculated using detrended and detrended-deseasoned mass anomalies and are given in Supplementary Figs. S3 and S4, respectively. These results reveal that the Sol1 provides significantly the best simulation and outperforms the previous studies when compared to CSRM within the GRACE/-FO period. For simplicity, we will use the name ‘ResDCAE’ to represent the DL model ‘Sol1’ in the rest of the paper.

### External Validation

#### Comparison with Greenland long-term surface mass balance estimates

In order to validate our simulation results, we performed a comparison with independent surface mass balance estimates of Greenland. Greenland is particularly chosen as a test bed because of the following three main reasons, among others. First, Greenland surface mass balance data is a unique independent data set which has a long temporal coverage, e.g., the IMBIE (Ice sheet Mass Balance Inter-comparison Exercise) surface mass balance data record starts ten years before the GRACE era. Second, the beginning of a dramatic increase in the ice mass loss trend was observed in ~2002^58^, almost right before the launch of GRACE mission. Thus, Greenland is the most challenging region on the Earth with a perfect data set to test the performance of the strategy adopted to mitigate the trend error in backwards extrapolation in this study. Third, as a consequence of the exacerbated global climate change, the melting of the Greenland ice sheets, and its peripheral glaciers and ice caps is the major contributor to contemporary sea level rise^59^.

Here, we use the most recent Greenland surface mass balance data from the IMBIE as the reference for comparison. The IMBIE data has been produced using 26 estimates of ice sheet mass balance derived from satellite altimetry (9 datasets), satellite gravimetry (14 datasets) and the input–output method (3 datasets) to assess changes in the Greenland Ice Sheet mass balance^27^. Prior to 2003, the estimates are solely from input-output method consistency of which with the estimates from satellite altimetry and gravimetry has been shown for common spatial and temporal domains after 2003 (see Fig. 2 of The IMBIE team^27^ for the number of individual mass balance estimates and the temporal coverage of the ach measurement type used). The final IMBIE surface mass balance data is available as a reconciled time series of cumulative total mass changes between 1992 and 2018 along with the uncertainty estimates and can be downloaded from http://imbie.org/data-downloads/.

**Fig. 4:**
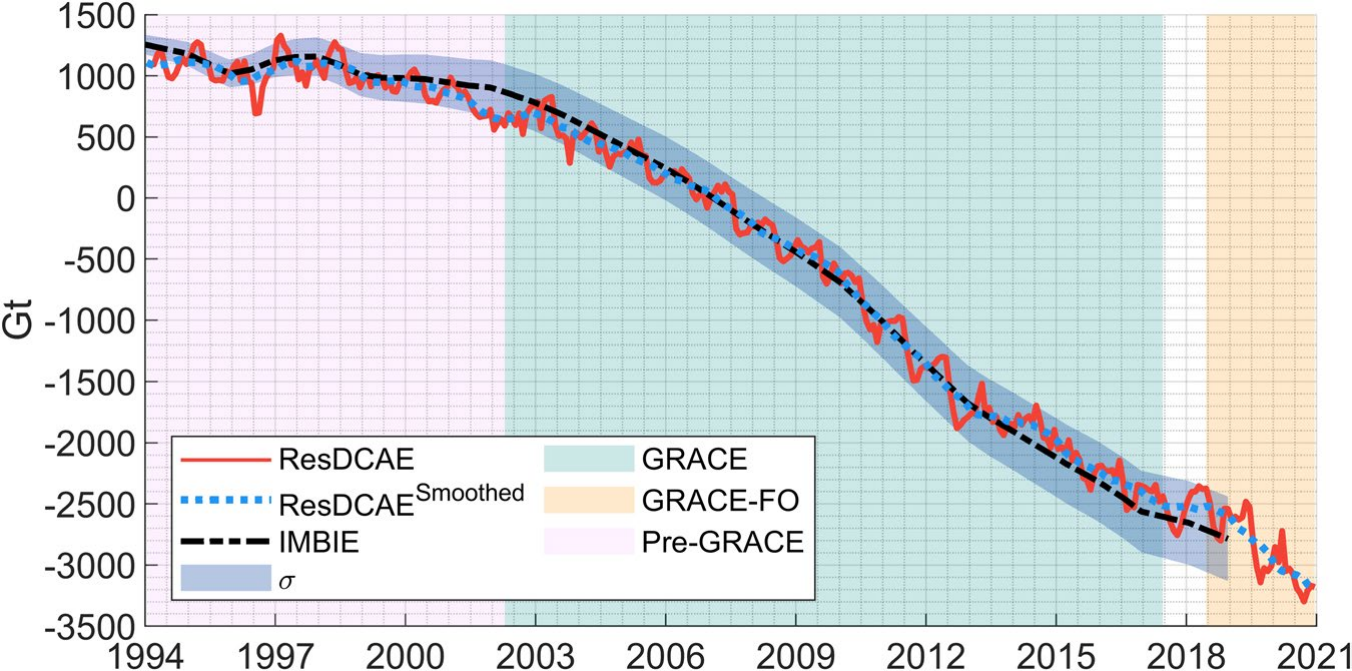
Cumulative mass change time series in Greenland from smoothed ResDCAE simulations (*dashed blue*) and from the IMBIE surface mass balance estimates (*dashed black*). Monthly mass anomaly from ResDCAE including seasonal mass change signal (*solid red*) is also shown. The shaded envelope represents the estimated 1-σ uncertainties of the cumulative changes of IMBIE.

The cumulative total mass change has been produced by integrating the rates of mass changes computed at annual intervals from time series of relative mass change using a 3-yr window. Therefore, for a fair comparison, we applied a 13-months moving average filter to our monthly mass change simulations from ResDCAE. The cumulative mass changes from the IMBIE surface mass balance and from ResDCAE (ResDCAE^Sm^°°^thed^) starting from 1994 are shown in Fig. 4. Note that the IMBIE data is referred to the mean baseline between 2004.000–2009.9999 to be consistent with the ResDCAE in the plot. The time series of original monthly ResDCAE mass change simulations as well as the estimated 1-σ uncertainties of the IMBIE are also presented in Fig. 4. Figure 4 shows an almost excellent agreement between IMBIE and the ResDCAE simulations throughout the entire time span. The slight differences between ResDCAE and IMBIE cumulative mass change time series are all within the 1-σ envelope of the IMBIE. The standard deviation is computed as ± 90 Gt based on these differences. The results indicate that our simulations are not only accurate within the GRACE/-FO era in which the training of the DL model was performed, but also provide good predictions of mass anomaly for the pre-GRACE era; the trend error mitigation strategy seems to work reasonably well even if not perfect.

#### Validation with ENSO events

The most active climate variability on the interannual timescale affecting long-term mass anomaly values is the El Niño–Southern Oscillation (ENSO), which results from large-scale ocean–atmosphere interactions over the equatorial Pacific^60–63^. Positive Sea Surface Temperature Anomalies (SSTA) in the eastern or central equatorial Pacific Ocean, as well as a weakening of equatorial trade, define the first phase of ENSO events, which occur every 2–7 years on average. El Niño events produce several severe droughts^64,65^ in the western Pacific and floods^66,67^ in the eastern Pacific, affecting climate globally. Besides that, the negative phase of ENSO is La Niña, which is a phenomenon in the tropical Pacific causing exceptional cooling of SSTs. The Southern Oscillation is the other part of ENSO, and it is a large-scale see-saw trend in the sea level pressure between the eastern and western tropical Pacific. El Niño results in low sea-level pressure in the eastern Pacific and higher pressure in the western Pacific, whereas La Niña has the reverse effect. Statistical models are frequently employed to determine ENSO evolution, with the SSTA index of Nino3.4 (120°W–170°W, 5°N–5°S, as given in Supplementary Fig. S5). These anomalies are the deviation of monthly SSTs from their long-term mean. When the Nino3.4 index surpasses + 0.5 °C and −0.5 °C for at least five consecutive months, it is considered an El Niño and a La Niña event, respectively. The 2015/16 El Niño is the most powerful event in recorded El Niño history. It exceeded the previous two extreme occurrences in 1997/98 and 1982/83^68,69^.

The ENSO has been demonstrated to have a significant impact on precipitation and air temperature in a variety of regions^70–72^. Mass anomaly is highly dependent on the integrated water mass changes due to precipitation, evapotranspiration, and runoff. It is also heavily influenced by regional meteorological circumstances such as droughts, flooding, and extended periods of high temperatures. All these components, particularly precipitation, are linked to ENSO. As a result, it is possible to conclude that ENSO and mass anomaly are related, as well. Several studies^66,70,73–75^ have demonstrated the impact of ENSO on mass anomaly in different basins of the Earth. For instance, Chen *et al*.^66^ explored the relationship between interannual mass anomaly variations and ENSO occurrences in the Amazon basin. Furthermore, Ni *et al*.^75^ analysed this phenomenon globally and discovered that ENSO occurrences have a significant impact on local Precipitation Anomalies (PPTA) and interannual TWS variations.

**Fig. 5:**
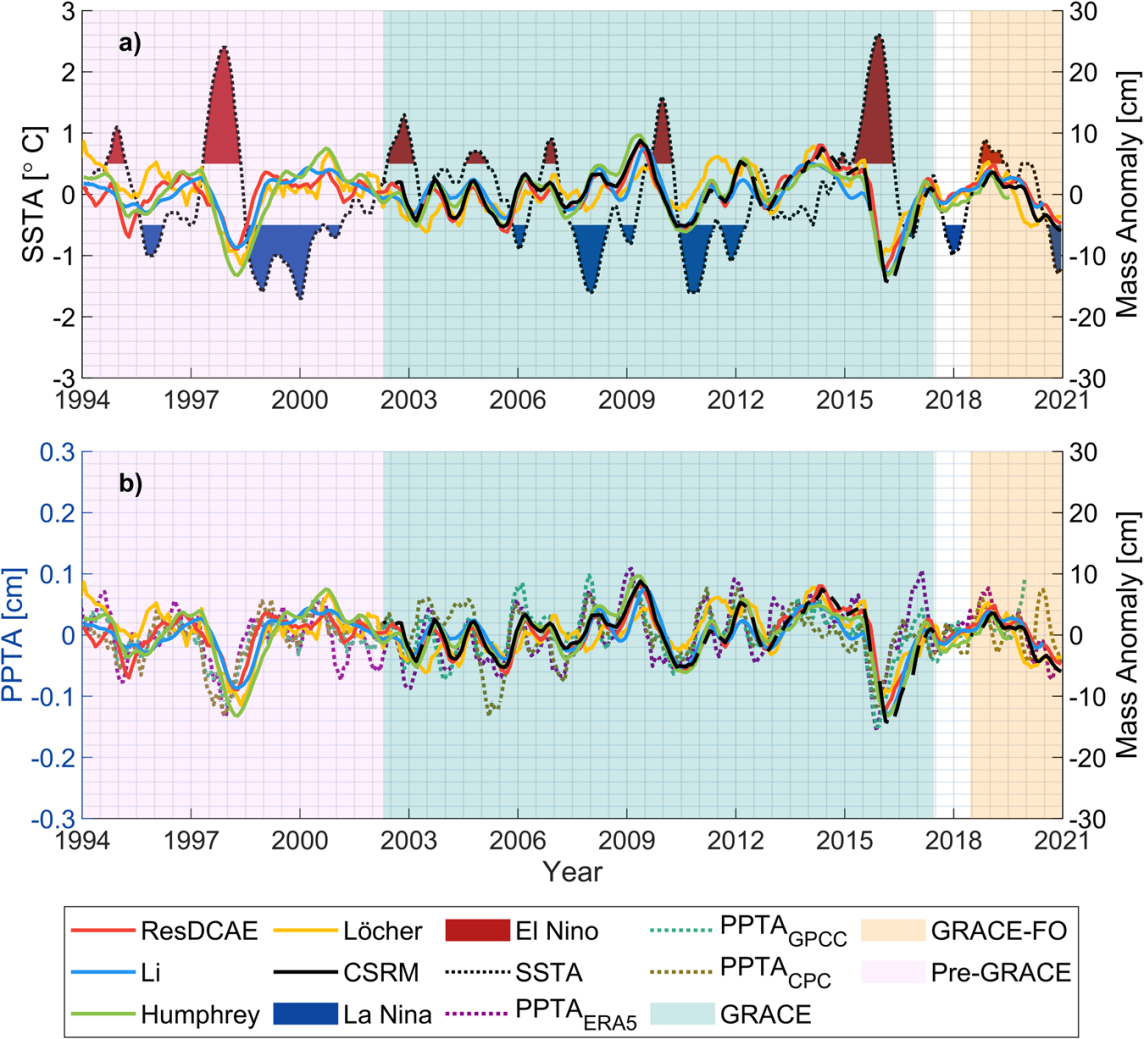
Time series of Nino3.4’s SSTA index (derived from the region 120°W–170°W, 5°N–5°S) and mass anomaly time series from previous studies, CSRM, and our simulation (ResDCAE) for the Amazon River basin calculated by averaging all grids in TRIP basin boundaries. (**a**) Comparison of the Amazon mass anomaly signal of all compared models to precipitation time series from ERA5, CPC, and GPCC datasets (**b**).

As the first external validation of our reconstruction, ResDCAE, a thorough assessment focused on the relationship between interannual mass anomaly and ENSO was carried out. It was also compared to previous studies which utilized the TRIP database for major river basin boundaries^44^. The mass anomaly reconstruction models are validated both with GRACE/-FO mass anomaly data and through examining their relationship with precipitation anomalies from ERA5, the National Oceanic and Atmospheric Administration (NOAA) Climate Prediction Center (CPC)^76^ and the Global Precipitation Climatology Center (GPCC)^77^ global precipitation dataset. The three different precipitation data above is chosen in order to ensure fair comparison among simulated mass anomaly from different studies. This is because ERA5 precipitation is an input dataset in both our ResDCAE and Humphrey while Li used CPC as an input for reconstruction. Furthermore, GPCC was also chosen due to its independence, regardless of the relationship between the input and simulations. Before the dataset used in validation, both CPC and GPCC were resampled to monthly temporal and 1.0° × 1.0° spatial resolutions. First, the average signals of reconstructions and precipitation datasets over river basins are calculated considering TRIP basin boundary data, which are also illustrated in Supplementary Fig. S5. In order to ensure consistency between these time series, 5-month moving average filters are applied as in the study by Ni *et al*.^75^, and then these smoothed time series are temporally matched to obtain the same time coverage for all datasets. According to Ni *et al*.^75^, the Amazon basin exhibits the highest correlation between mass anomaly and ENSO events, according to their global analyses, and it takes 5 months for the influence to appear in the basin. The illustration of average basin signals for the Amazon basin is given in Fig. 5. In Fig. 5a,the Nino3.4 index (http://www.cpc.ncep.noaa.gov/data/indices/) is employed as a measure of ENSO activity in a comparison to simulated mass anomaly in the Amazon basin. A similar comparison of simulated mass anomaly versus precipitation anomalies from three different precipitation data set is also given in Fig. 5b. Figure 5a,b show that interannual mass anomaly variations are strongly linked to ENSO and precipitation with several months of time lags over the Amazon basin, particularly during massive El Niño events in 1997/98 and 2015/16. Furthermore, when the time series of the models are compared to CSRM, it is found that the ResDCAE model distinguishes rapid changes more easily and matches well with the CSRM mass anomaly. The GRACE and pre-GRACE correlations between the time series of all investigated mass anomaly solutions and the SSTA time series are calculated separately. The calculated correlations are all maximal values with specific phase lags. The correlations for the comparison between ResDCAE and SSTA in the GRACE-period were −0.57 (6-month lags) and −0.85 (4-month lags) in the pre-GRACE period. Similarly, the correlations for the Li, Humphrey, Löcher, and CSRM time series were calculated as −0.65 (5), −0.73 (6), −0.60 (7), and −0.57 (5) for the GRACE period, whereas they (excluding CSRM) were calculated as −0.87 (6), −0.84 (7), and −0.63 (5), respectively for the pre-GRACE period. During the GRACE period, our ResDCAE solution has nearly the same correlation as the CSRM time series. This results in a significant convergence to the simulated CSRM data. On the other hand, our simulation is also consistent with other studies in both time periods.

**Fig. 6:**
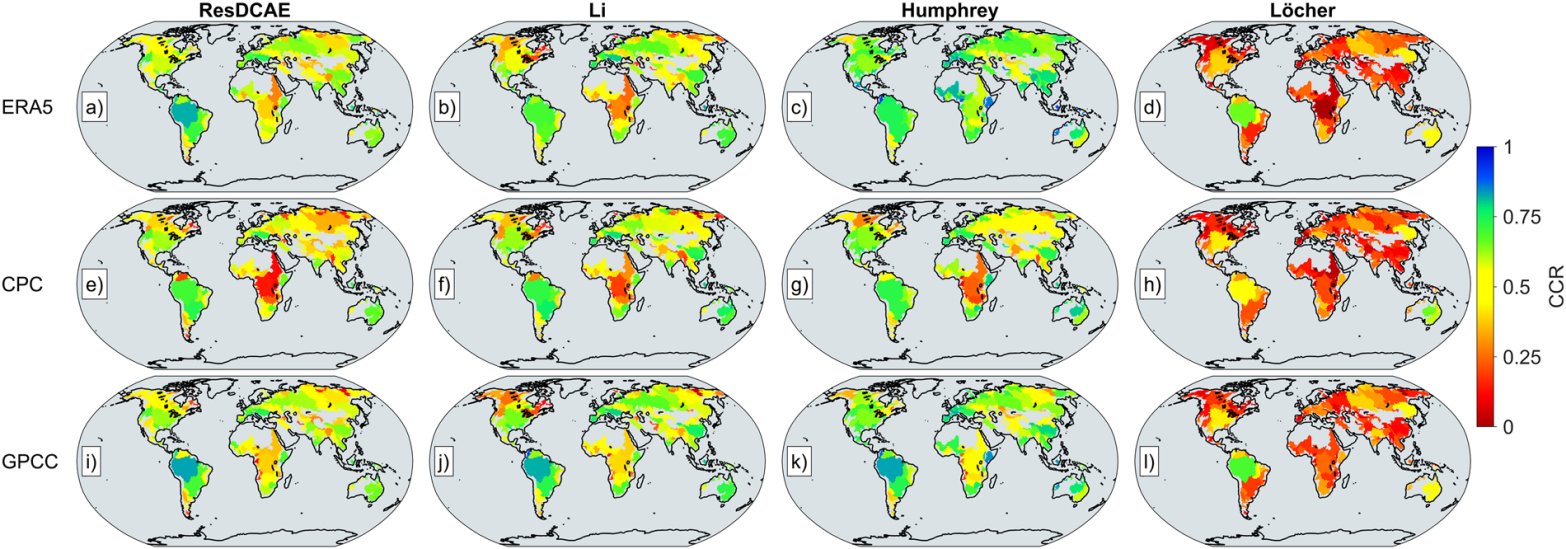
Cross-correlations between precipitation anomalies, from (*top to bottom*) ERA5, CPC, GPCC and mass anomaly simulations from (*left to right*) ResDCAE (**a,e**, and **i**), Li (**b,f**, and **j**), Humphrey (**c,g**,and **k**) and Löcher (**d,h** and **l**) at 176 river basins defined in the TRIP database, respectively.

The time series of all three precipitation anomaly data are in good agreement with the mass anomaly simulations. In order to quantify the Cross-Correlations (CCR) between these precipitation anomalies and mass anomaly simulations at all river basins in the TRIP database, the CCR coherence spectrum is calculated by determining and considering time lags between all signals separately. Totally 176 river basins are considered in this comparison. Each comparison, e.g., ResDCAE vs ERA5 PPTA for all basins, has its own defined time lag, which is determined by the maximum correlation computed between the compared time series. Thus, both cross-correlations and time lags are calculated for all compared time series pairs at each river basin. CCR metrics are given in Fig. 6a–l for the comparison between mass anomaly simulations and ERA5, CPC, and GPCC datasets. The lags between the time series of mass anomaly simulations and precipitation are also given in Supplementary Fig. S6. According to Fig. 6, almost similar CCR are calculated between all mass anomaly simulations and precipitation datasets, except for Löcher’s. The discrepancies between precipitation and mass anomaly simulations of Löcher is most likely due to the fact that, contrary to ResDCAE, Humprey and Li, no precipitation data was considered when Löcher’s spherical harmonic coefficient models are calculated. On the other hand, as expected, Humphrey’s simulations have slightly higher correlations with PPTA in all basins, because the precipitation data has been directly used as the dominant input in a linear water store model (see Eq. 1 of Humprey and Gudmundsson^6^). Nevertheless, all simulations have similar CCR in almost all river basins. As it can be seen in Fig. 6a–l, the Amazon River basin has the highest CCR (>0.75) in all comparisons.

#### Validation with independent degree-2 spherical harmonic coefficient estimates

The long-wavelength components of gravity change due to variations of mass redistribution on Earth can also be recovered from SLR tracking measurements or EOP, independently^48,78^. Thanks to advancements in satellite geodetic techniques, EOP- and SLR-derived low-degree spherical harmonic coefficients, i.e., ∆C_20_, ∆C_21_ and ∆S_21_, can be determined with higher accuracy than GRACE/-FO observations^48^. These degree-2 coefficients are related to the different geophysical dynamics of mass redistribution on Earth^1,48^. These relationships could be exemplified by the fact that while SLR-derived ∆C_20_ provides information about mass variations due to the oblateness of the Earth, ∆C_21_ and ∆S_21_ are related to the variations of the Earth’s rotational axis^79,80^. Therefore, degree-2 spherical harmonic coefficients recovered from GRACE/-FO could be validated independently using EOP- and SLR-derived counterparts.

In order to validate our simulations, global 1.0° × 1.0° gridded mass anomaly from ResDCAE were first converted to spherical harmonic coefficients using Eq. 35 of Wahr *et al*.^81^. The maximum degree and order of the spherical harmonic expansion were chosen as 96. Note that these spherical harmonic coefficients represent the relative anomalies w.r.t the 2004.0–2009.9999 mean baseline, i.e., the coefficients are ∆C_nm_ and ∆S_nm_. Similarly, CSRM mass anomaly were also converted to spherical harmonic coefficients (CSRM spherical harmonic coefficients). On the other hand, monthly C_20_ coefficients estimated from SLR were taken from https://grace.jpl.nasa.gov/data/get-data/oblateness/ and the 2004.0–2009.9999 mean was removed from the entire time series to calculate the SLR ∆C_20_ series consistent with those of ResDCAE and CSRM. Note that the background models adopted within CSRM and CSR GRACE/-FO RL06 processing chain have also been used for the recovery of SLR-only gravity field models for comparison in this study^78,79^. Further, EOP-derived, ∆C_21_, ∆S_21_, and ∆C_20_ coefficients were calculated from the mass term of the Earth Rotation excitations using Eq. 2 of Chen *et al*.^82^. Mass excitations are obtained by subtracting motion terms from observed excitations of polar motion components (X, Y), and Length of Day (LOD), respectively. While the mass excitations are due to the mass load variations, the motion excitations arise from the angular momentum exchange between the Earth’s crust and the atmosphere, i.e., due to the frictions of atmospheric wind and ocean current fields on the Earth^82,83^.

**Fig. 7:**
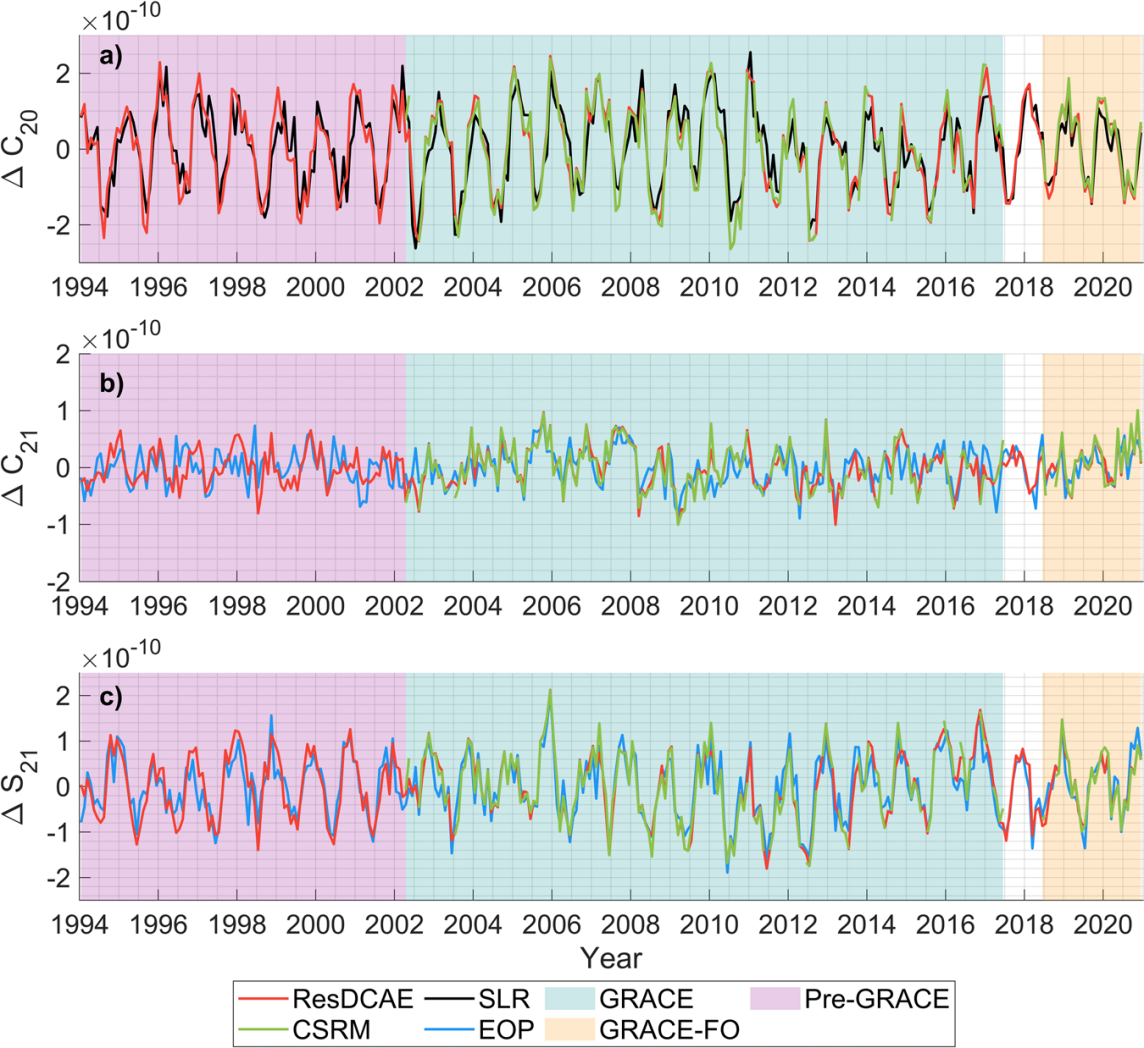
Comparison of ResDCAE-, CSRM-, EOP- and SLR-derived low-degree spherical harmonic coefficients, (**a**) ∆C_20_, (**b**) ∆C_21_ and (**c**) ∆S_21_.

Daily Polar motion (X, Y) and LOD observations are taken from the International Earth Rotation and Reference Systems (IERS) EOP 14 C04 series^84^. In order to calculate mass terms, the daily observed and motion excitations were computed using interactive tools of the IERS (https://hpiers.obspm.fr/eop-pc/analysis/excitactive.html) with the Chandler period set at 433 days and quality factor at 100. The motion terms are also computed considering the angular momentum series, namely ECWMF and Max-Planck-Institute for Meteorology Ocean Model (MPIOM), that are provided by GFZ^85^ which also serve as a basis for atmosphere and ocean de-alising (AOD1B RL06) model in GRACE/-FO data processing^83^. Thus, the consistency between ResDCAE and EOP-derived also ensured using these models as angular momentum components of motion terms. The daily mass excitations are obtained by removing motion terms from observed excitations and these datasets comprise different periodic signals, such as the 5.8-yr oscillation in the observed LOD series, which is sourced from core-mantle interaction and is not related to gravity change^86^. Thus, first a zero phase-shift Butterworth high-pass filter with a cut-off frequency of 1/4 cpy is applied to remove this oscillation and other long-period signals from the LOD series. Besides, the linear trends were removed from all mass excitations using unweighted least squares trend estimation because the long-term variation was in good agreement with the EOP- or SLR-derived series at seasonal time scales. After that, the daily degree-2 coefficients were computed using these detrended mass excitations, and a low-pass filter with a cut-off frequency of 6 cpy was applied to remove from the every signal shorter than 2 months. Finally, the monthly ∆C_21_, ∆S_21_, and ∆C_20_ were computed by averaging the daily ones in each month. In order to make a fair comparison, the corresponding time series of ResDCAE, CSRM and SLR were also detrended using unweighted least squares. All the detrended series are presented in Fig. 7.

All degree-2 coefficient time series are in a good agreement of ± 2e-10 for both GRACE and GRACE FO period (Fig. 7). In contrast to ∆C_21_ and ∆S_21_, EOP-derived ∆C_20_ has a phase-shift of about 4-months that may be sourced from high-pass filtering applied to the remove the long-period signals from ∆LOD, and therefore not shown in Fig. 7a. On the other hand, the seasonal amplitude of ∆S_21_ series is higher than that of ∆C_21_. This is because ∆S_21_ is more sensitive to mass changes over land while ∆C_21_ is more sensitive to mass changes over the oceans. Similar to the results in e.g., Meyrath *et al*.^86^ and Chen *et al*.^82^, while GRACE/-FO-derived (ResDCAE and CSRM) ∆C_20_ coefficients are more consistent to those estimated from SLR ones, the ∆C_21_ and ∆S_21_ from ResDCAE and CSRM are both closer to each other and the EOP-derived estimates. Zero phase-lag correlations, after removing annual/semiannual variations and linear trends using unweighted least squares fit, between every pair of estimates of ∆C_20_, ΔC_21_ and ΔS_21_ shown in Fig. 7 are listed in Table 2. As expected, for all three coefficients, the highest correlations (0.94-0.96) are observed between ResDCAE and CSRM estimates when compared to others within the GRACE/-FO era. The correlations between the ∆C_20_ from ResDCAE and from SLR within and pre-GRACE era are still as high as 0.80 and 0.70, respectively. The correlations for ∆C_21_ are slightly smaller than those for ∆S_21_ in all comparisons, which may be due to the lower signal-to-noise ratio of ∆C_21_ as it is more relevant to the mass change signal over the oceans. The correlations given in Table 2 within GRACE/-FO era are all comparable to the results from earlier studies, e.g., Chen *et al*.^83^, Meyrath *et al*.^86^ although they used the coefficient estimates from GRACE/-FO Level-2 GSM data rather than mascon solutions. This, as well as the different time spans used to compute correlations explain the slight differences between their results and the results in this study. It is worth noting that the degree-2 coefficients from our ResDCAE model and from GRACE/-FO are converted from mascon type mass grids on Earth. The coefficients from ResDCAE model simulations also reveal reasonably high correlations (0.58 for ∆C_21_ and 0.65 for ∆S_21_) with EOP-derived ones before the GRACE era which shows the efficiency of the methodology (DL + backwards trend error mitigation strategy) used in this study.

#### Validation with global barystatic mean sea level change data

Barystatic mean sea level change data is another independent data source used to validate GRACE/-FO temporal gravity solutions^48^ on global scale. Satellite altimetry has been a well-established space geodetic technique for accurately measuring global sea level change for about three decades. Thus, we compared our simulation results over the oceans with the altimeter-observed Global Mean Sea Level (GMSL) change time series after appropriate preprocessing steps were applied. To this end, the time series of GMSL anomalies (from altimetry) and associated steric components are calculated and compared to mean ocean mass change retrieved from ResDCAE simulations and CSRM. GMSL records consist of both ocean mass and steric components and can be expressed with the sea level-budget equation as GMSL_altimetry_ = GMSL_steric_ + GMSL_oceanmass_. While GMSL_steric_ represents the contributions of oceans’ thermal expansion and salinity to sea-level variations, GMSL_oceanmass_ represents the change in ocean mass^87,88^. Before comparing the time series of GMSL to ocean mass anomalies from ResDCAE and CSRM, GMSL_steric_ must be removed from the GMSL_altimetry_ anomalies to obtain GMSL_oceanmass_ in order to make physically consistent comparison.

The GMSL_altimetry_ dataset is provided by Copernicus Marine Environment Monitoring Service (CMEMS) and the Copernicus Climate Change Service (C3S) and derived from ECWMF database^89,90^, which includes the gridded (with daily and 0.25° × 0.25° spatial resolutions) merged satellite altimetry sea level anomaly data products combining different satellite altimetry observations and covering the time span from January 1993 to present. We downloaded the gridded GMSL_altimetry_ dataset from ECWMF from January 1994 to December 2017. We confined our comparison between this time span as there is no reliable data available for computation of steric contribution beyond December 2017^91^. Some pre-processing steps such as upsampling and some corrections, were applied before the calculating GMSL_altimetry_ time series from gridded dataset. First, GMSL dataset were upsampled to monthly 1.0° × 1.0° grids by averaging from its own native resolutions to ensure consistency with our simulations. After that, the so-called TOPEX-A instrumental drift corrections, which is sourced from the instrumental problems of satellite and spanning the period from January 1993 to December 1998^87^, were added to each grid using provided correction values along with the dataset. On the other hand, GMSL_steric_ component was calculated from gridded steric-height anomalies that are retrieved from Camargo *et al*.^92^, which is the ensemble mean derived from the 10 different temperature and salinity data sets and has monthly sampling with 1.0° × 1.0° spatial resolution covering oceans between 66° N–66° S latitudes from January 1993 to December 2017.

GMSL_altimetry_ and GMSL_steric_ anomalies are calculated considering the mean baseline between 2004.0 and 2009.9999 to ensure consistency with ocean mass change from our simulations as well as from CSRM. Then the time series are obtained by averaging grids over the oceans, excluding a 300-km buffer zone along the coasts to avoid any signal leakage from land hydrology. The average monthly sampling of time series was obtained from the weighted ocean mass change grids. The weights were determined considering the surface area of each grid cell over the oceans within 65° N–65° S latitudes. In addition, GIA correction was applied to GMSL time series by adding a constant value of −0.23 mm/year derived from the ICE-6G_D VM5a model^39^, which also was used as GIA correction CSRM. GMSL_oceanmass_ time series was then calculated by removing GMSL_steric_ from GMSL_altimetry_ time series to compare with ocean mass change from ResDCAE simulations and CSRM. The same averaging procedure was also applied to the time series of ResDCAE simulations (from January 1994 to December 2017) and CSRM (from April 2002 to December 2017). The GIA and GAD corrections are readily included in our simulation as it uses the corrected version of CSRM as output data for training the DL model. Finally, the seasonal (annual/semi-annual) signals were removed using unweighted least-squares from all time series and a moving average filter with a window length of 400 days was applied to each of the time series before comparison.

**Fig. 8:**
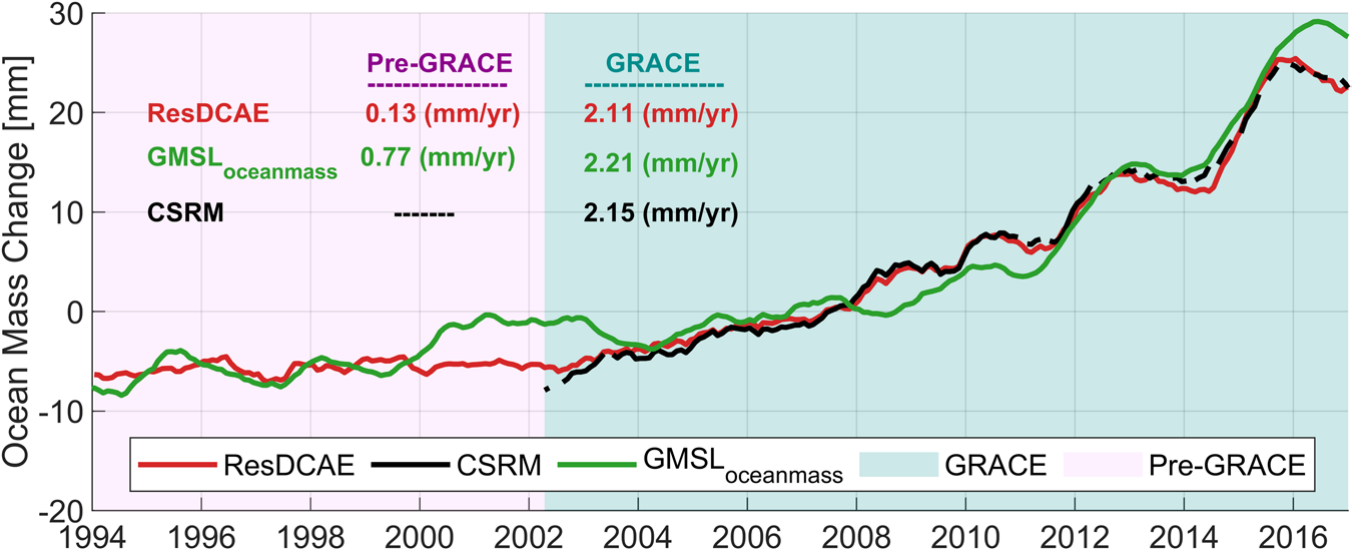
Deseasoned global mean ocean mass change time series (in mm equivalent water height) from our ResDCAE simulation (*solid red*), from steric corrected altimetry (*solid green*) and from original CSRM computed over the ocean grids between 65° N and 65° S latitudes. The altimetry derived GMSL time series (*dashed blue*) is from Horwath *et al*.^94^ and the steric component (*dashed yellow*) is from Camargo *et al*.^92^ both are presented for completeness.

Ocean mass change time series both from our ResDCAE simulation and CSRM as well as the altimetry derived GMSL_oceanmass_ are given in Fig. 8. While trend values are calculated for GRACE period as 2.11, 2.15, and 2.21 mm/yr, they are calculated for pre-GRACE period only for ResDCAE and GMSL_oceanmass_ as 0.13 and 0.77 mm/yr shown in Fig. 8. The long-term linear trends estimated from ResDCAE, CSRM, GMSL_altimetry_, GMSL_steric_ and GMSL_oceanmass_ time series are 1.47, 2.15, 2.82, 1.47 and 1.34 mm/yr, respectively. The deseasoned time series of ocean mass change are consistent to each other especially after 2004. This improvement can be attributed to the accurate Argo-based steric height models developed in early 2005^87,93,94^. Dieng *et al*.^87^ has shown that the ensemble members of steric heights used in various studies show significant differences between 1993 and 2004. However, Argo data from January 2005 to end of 2015 significantly reduce the uncertainties of the steric sea level change data products^87,94^. We re-estimated the linear trends from all time series but for the time period from January 2005 to December 2017 and obtained 2.26, 2.26, 3.58, 0.88 and 2.70 mm/yr, respectively for ResDCAE, CSRM, GMSL_altimetry_, GMSL_steric_ and GMSL_oceanmass_. The GRACE-based (ResDCAE simulations and CSRM) ocean mass change time series and altimetry-steric (GMSL_oceanmass_) are all in excellent agreement throughout the Argo data time-span. We also computed the correlations of the original (non-detrended/non-deseasoned) ResDCAE simulations as well as of the CSRM with the Altimetry-Steric (barystatic) sea level change time series. Within the GRACE era (April 2002 – December 2017) a correlation coefficient of 0.86 is obtained both with ResDCAE and CSRM. The correlation coefficient computed between ResDCAE and the Altimeter-derived barystatic sea level for the pre-GRACE era (January 1994 – March 2002) is still as high as 0.79, indicating reasonably well simulation performance and the effectiveness of the trend error mitigation strategy (c.f. section Methods) adopted in this study.

#### Validation with *in situ* ocean bottom pressure data

The simulated mass changes over oceans can be compared to *in situ* ocean bottom pressure (OBP) observations for qualitative validation. The detection of spatiotemporal mass variations over oceans from GRACE/-FO is more challenging than those of the continental hydrology signal since the detectable variations of gravity signal over the ocean are weaker^95^. Moreover, the comparison of these variations to independent point-wise OBP variations is much more challenging due to the differences between spatial and temporal resolutions, the irregular distribution of OBP stations over the globe, or the necessity of isolating signals that are related to ocean circulation and sea level changes^96^. Considering these aspects, *in situ* OBP observations that are publicly available in the Permanent Service for Mean Sea Level (PSMSL) database (https://www.psmsl.org/data/bottom_pressure/) were used to compare to the gridded mass change time series from our simulation and CSRM. To this end, we chose two stations from PSMSL database considering the temporal coverage of data records (stations with data records available for longer time) and taking into account the proximity and distance from the land to test any possible leakage effect from the land. Thus, the stations Dark Passage South – DPS (60.9° S–54.7° W) and NDBC 51406 – Central South Pacific (8.5° S–125.0° W) were chosen the start and end deployments of which are November 1992 – June 2011 and September 2001 – February 2013, respectively.

**Fig. 9:**
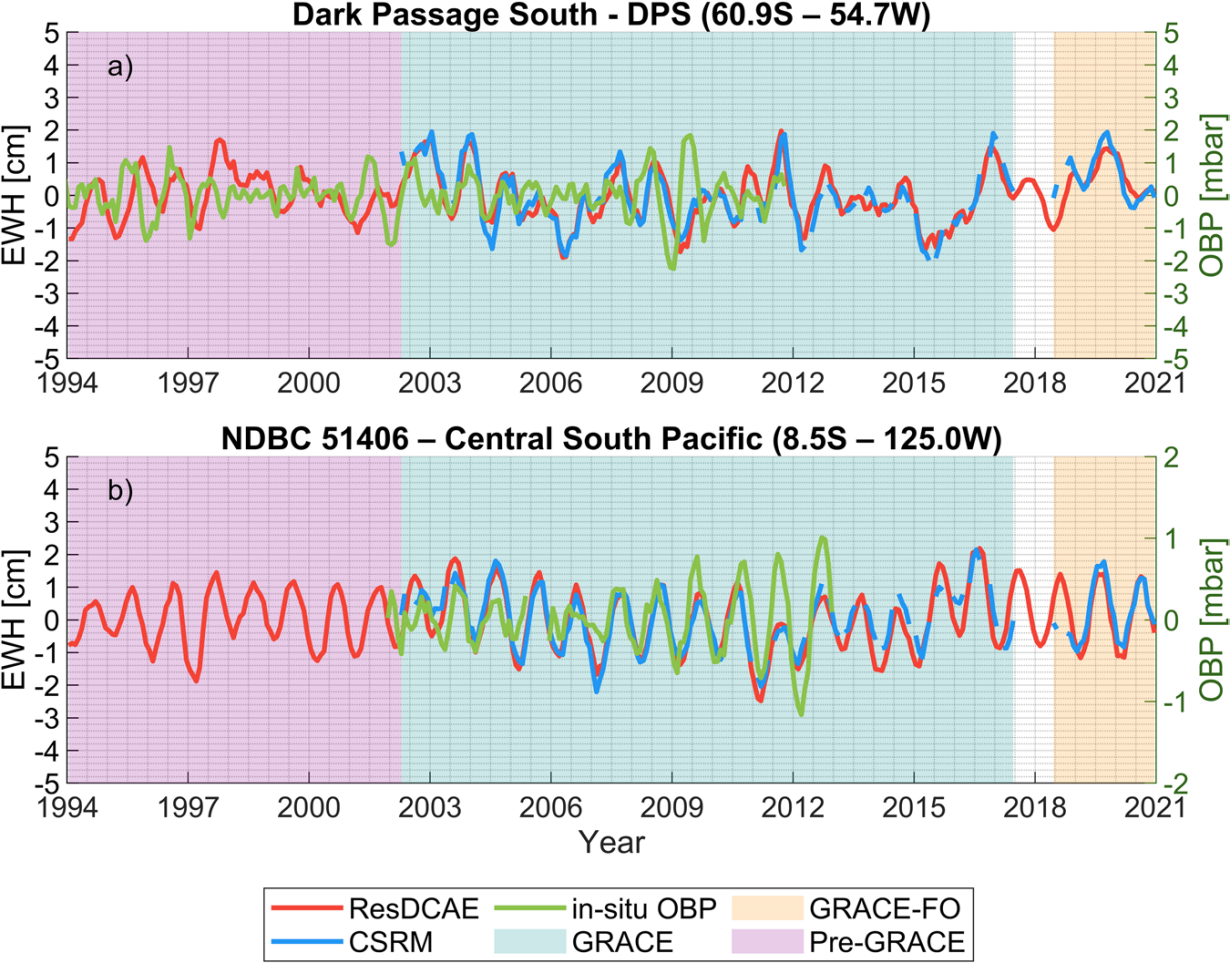
Time series comparisons of *in situ* OBP measurements (*green*) with mass anomaly data from ResDCAE (*red*) and CSRM (*blue*) at two selected stations: (**a**) Dark Passage South – DPS (60.9° S – 54.7° W) and (**b**) NDBC 51406 – Central South Pacific (8.5° S – 125.0° W). The mass anomaly plots represent the monthly values at the 1° × 1° grid which contains the location of the corresponding OBP station.

The daily sampled mean OBP data constructed from hourly observations at each deployment are readily available after removal of diurnal and shorter period tides by averaging 24 hourly values and sensor drift corrections. The long-term trends as well as the remaining drifts were first removed with a quadratic fit from daily OBP time series at each deployment as suggested by Poropat *et al*.^96^ *in situ*. In order to generate monthly sampled OBP time series, first the low-pass Butterworth filter was applied with 12 cpy cut-off frequency to remove any remaining signal with sub-monthly periods from the daily time series. The monthly time series were then computed by averaging these filtered daily samples. On the other hand, the monthly mass change time series from ResDCAE and CSRM at the two 1.0° × 1.0° grids which contain the selected OBP stations were first detrended. The monthly time series of *in situ* OBP and mass change usually need to be compared for long-wavelength signal content due to the complexity between observed mass change by GRACE and OBP induced by their differences in spatial and/or temporal resolutions. Therefore, six-months moving average filter was further applied to monthly sampled ResDCAE and CSRM time series. The resulting time series are given with a dual axis plot in Fig. 9.

The agreement between *in situ* OBP and mass change time series is better at NDBC station than at DPS station as shown in Fig. 9a,b. This is most likely due to the signal leakage from land and sea ice at northern Antarctica to the mass change signal at DPS station. The NDBC station, on the other hand, is in the open ocean and thus almost no signal leakage from land hydrology exists. The comparison of GRACE-based mass change solutions to *in situ* OBP observations is highly dependent on both oceanographic priors and applied post-processing as well as the basin size adopted for averaging while generating the mass change time series^96,97^. Despite the fact that the general signal preprocessing tools such as filtering and smoothing were applied to obtain time series of ResDCAE, CSRM, and OBP observations, the agreements with *in situ* OBP records at these two different locations over the oceans are comparable with those in previous studies^98,99^.

### Summary and Future Perspectives

In this study we employed a hybrid deep learning architecture called resDCAE to simulate mass anomalies at a spatial resolution of 1.0 degree by 1.0 degree and a monthly temporal resolution from January 1994 to January 2021. We proposed and successfully performed a strategy to reduce the error of the trend component in the simulations during the pre-GRACE period (1994 to 2002). The primary objective was to achieve a better understanding and characterization of various climate-induced geophysical phenomena, including the terrestrial water cycle, ice sheet and glacier mass balance, sea level changes, and variations in ocean bottom pressure by providing longer time series of global water storage changes both over continents and oceans. The research demonstrated that the use of a combination of ERA5 and SLR datasets, along with time channel information, provided better, if not the best, solution for simulations. This study contributes to the monitoring and comprehension of long-term global gravity field changes, offering valuable insights into climate change and other significant geophysical events. Such research is advancing our understanding of climate changes and their impacts on Earth’s water cycle. With the new data sets as well as advanced satellite gravity missions and developments in deep learning era and algorithms, improved simulations of the water mass change with enhanced resolutions will be possible in the future.

## Usage Notes

The simulated data is available with no gaps from January 1994 to end of December 2020 both in the form of monthly 1.0° × 1.0° mass anomaly grids and spherical harmonic coefficients similar to official GSM data products but with a much higher resolution up to degree and order 200. The user should note that the data set may not include seismic signal and thus is not proper for e.g. earthquake signal detection. For conversion of mass anomaly grids to spherical harmonic coefficients, Equations 6–8, and load Love numbers in Table 1 of Wahr *et al*.^81^ were used with ρ_ave = _5517 kg/m^3^ as the average density of the solid Earth. Both data sets represent the anomalies relative to 2004.0–2009.9999 mean baseline similar to CSR mascon solutions. When using spherical harmonic coefficients data, no further destriping or smoothing filter is required.

### Supplementary information

41597_2024_2887_MOESM1_ESM

## Data Availability

There is no customized code in generation or processing of datasets. For setting up and training the Deep Learning Models, the publicly available codes in Python language from TensorFlow^9^ and Keras^10^ libraries were used. The trend error mitigation and all the figure plots in the paper were implemented using the existing routines/functions in MATLAB software.
