# Supplementary material for: High-resolution temporal gravity field data products: Monthly mass grids and spherical harmonics from 1994 to 2021
Source: Sci Data. 2024 Jan 13;11:71. doi: 10.1038/s41597-023-02887-5 (PMC10787793; doi:10.1038/s41597-023-02887-5)
Supplement: Supplementary file 1 — 41597_2024_2887_MOESM1_ESM [file 41597_2023_2887_MOESM1_ESM.pdf]

Supplementary Information for

High-resolution temporal gravity field data products: Monthly mass grids and spherical harmonics from 1994 to 2021

Metehan Uz[1], Orhan Akyılmaz[1✉], C K Shum[2], Kazım Gökhan Atman[3,4], Sevda Olgun[5], Özge Güneş[6]

[1] Dept. of Geomatics Eng., Istanbul Technical University, Istanbul, Turkey

[2] Division of Geodetic Science, School of Earth Sciences, The Ohio State University, Columbus, Ohio, USA

[3] School of Mathematical Sciences, Queen Mary University of London, London, England

[4] Department of Physics, Ege University, Izmir, Turkey

[5] Dept. of Geomatics Eng., Kocaeli University, Kocaeli, Turkey

[6] Dept. of Geomatics Eng., Yıldız Technical University, Istanbul, Turkey

✉ Correspondence to: Orhan Akyılmaz (akyilma2@itu.edu.tr)

Content

Supplementary Figures S1-S6……………………………………………………………......1

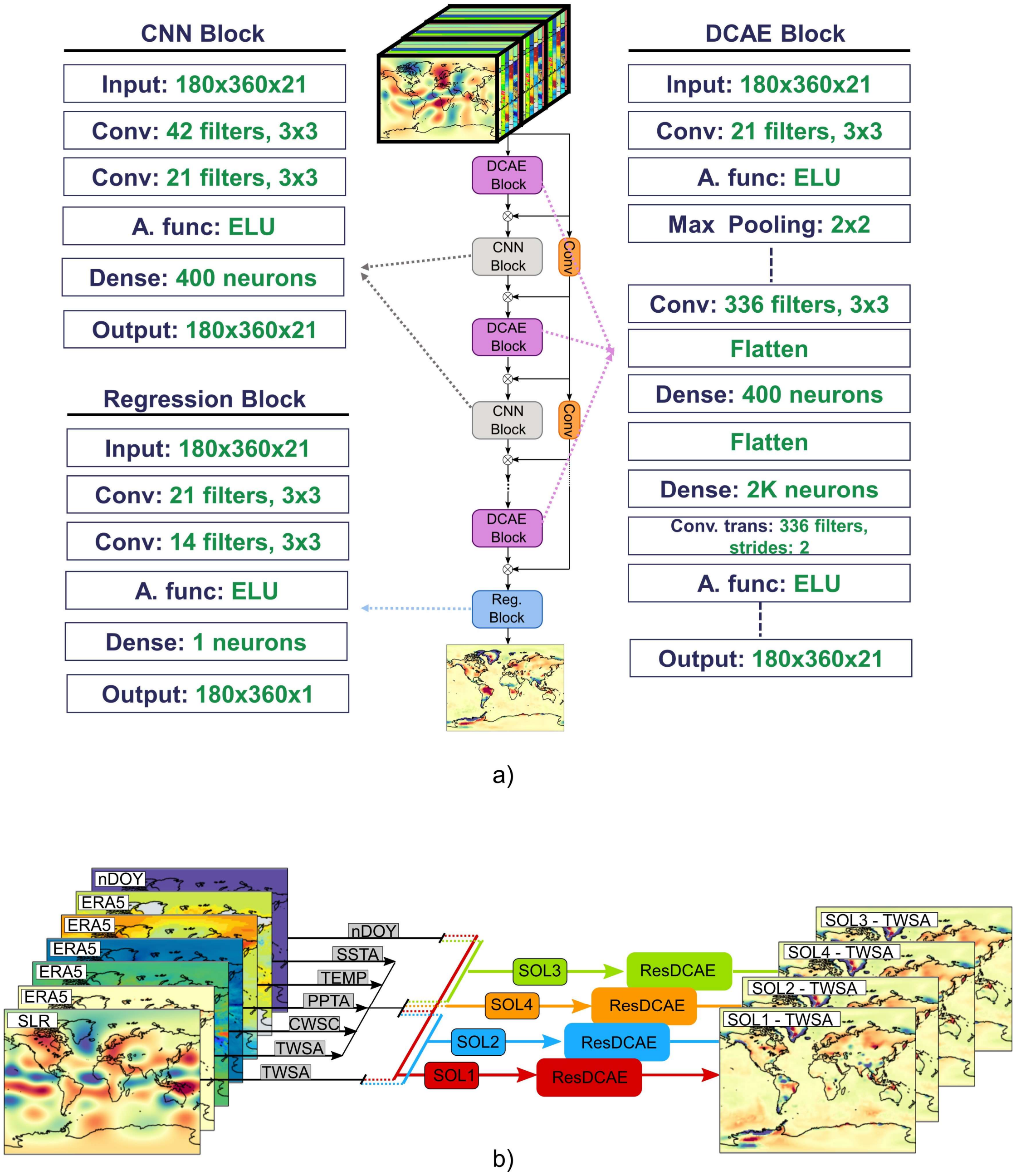

Fig. S1 The architecture of the proposed ResDCAE model

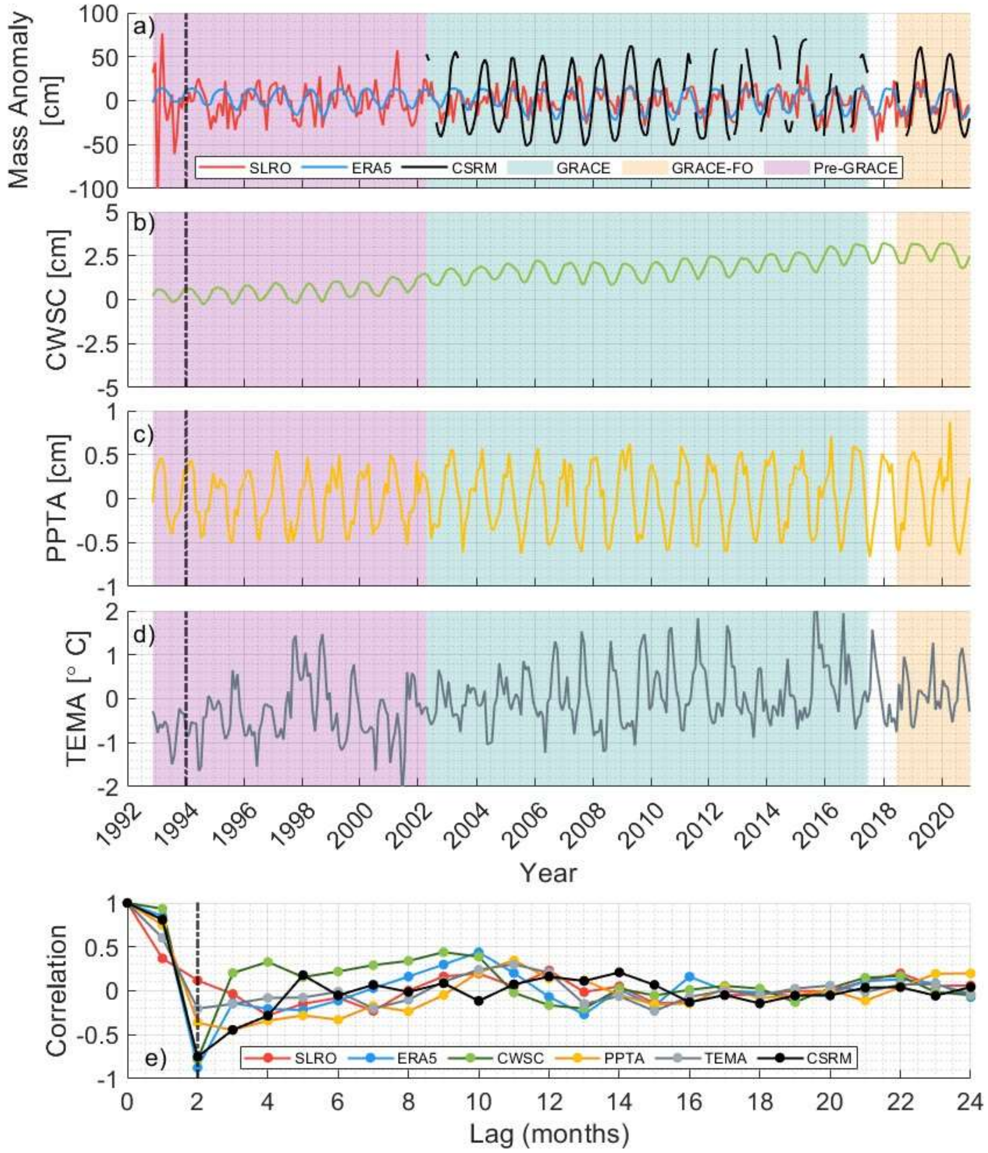

Fig. S2 Time series of each input and output data at the grid located at the centre of Amazon basin (a-d), and partial autocorrelation results calculated for each of the time series (e).

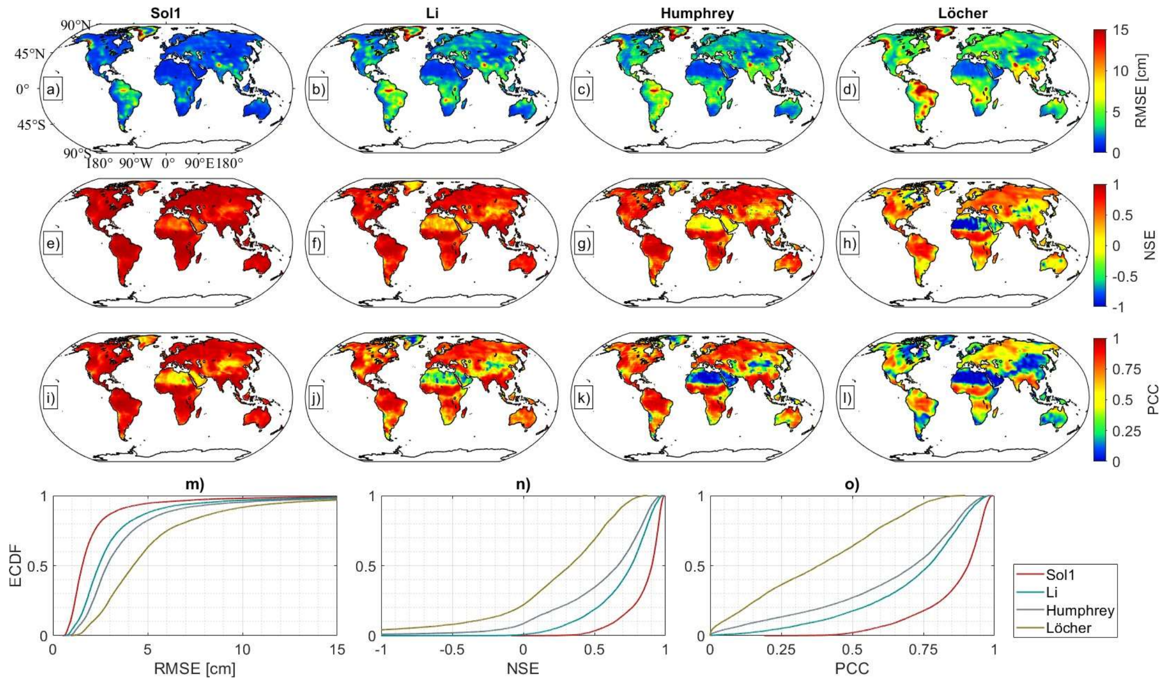

Fig. S3 The same with Fig. 3, but computed after detrending for (a, e, i) Sol1, (b, f, j) Li et al.[1], (c, g, k) Humphrey and Gundmundsson[2] and (d, h, l) Löcher and Kusche[3], respectively using 175 common months of TWSA solutions from all four studies, and the corresponding ECDF of (m) RMSE, (n) NSE and (o) PCC.

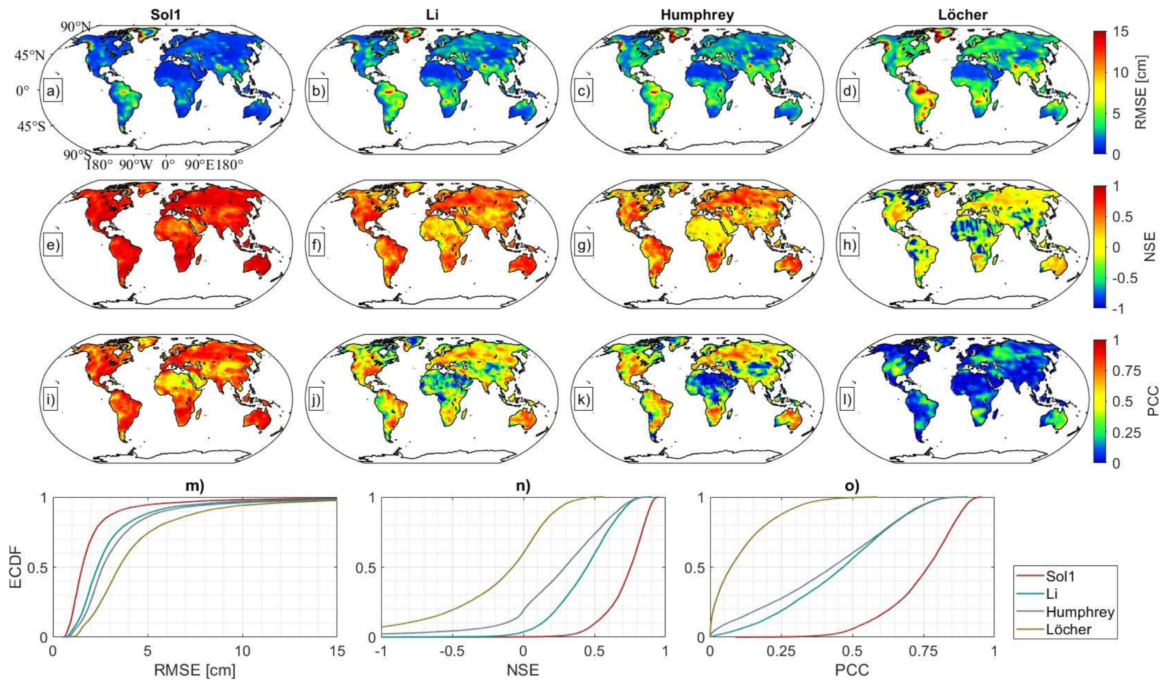

Fig. S4 The same with Fig. 3, but computed after detrending and deseasoning, for (a, e, i) Sol1, (b, f, j) Li et al.[1], (c, g, k) Humphrey and Gundmundsson[2] and (d, h, l) Löcher and Kusche[3], respectively using 175 common months of TWSA solutions from all four studies, and the corresponding ECDF of (m) RMSE, (n) NSE and (o) PCC .

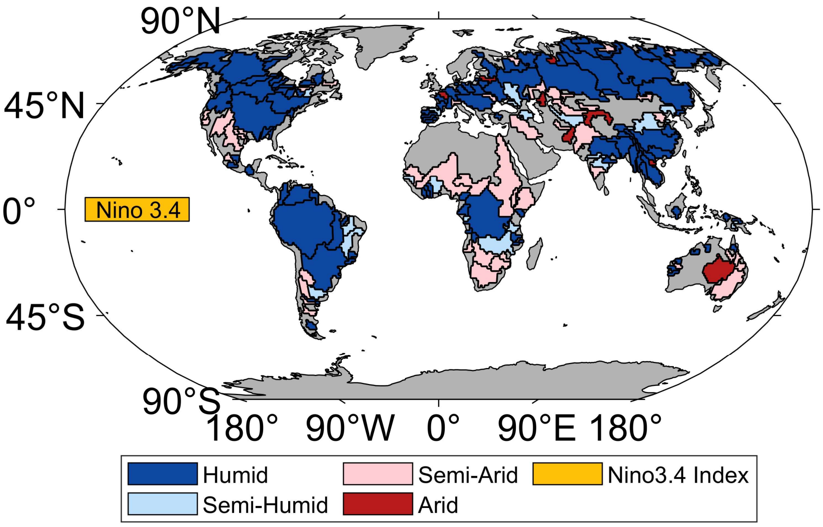

Fig. S5 Illustration of Total Runoff Integrating Pathway (TRIP) basin boundaries[4] w.r.t. the climatic conditions and region of Nino3.4 SSTA index (120° W–170° W, 5° N–5° S).

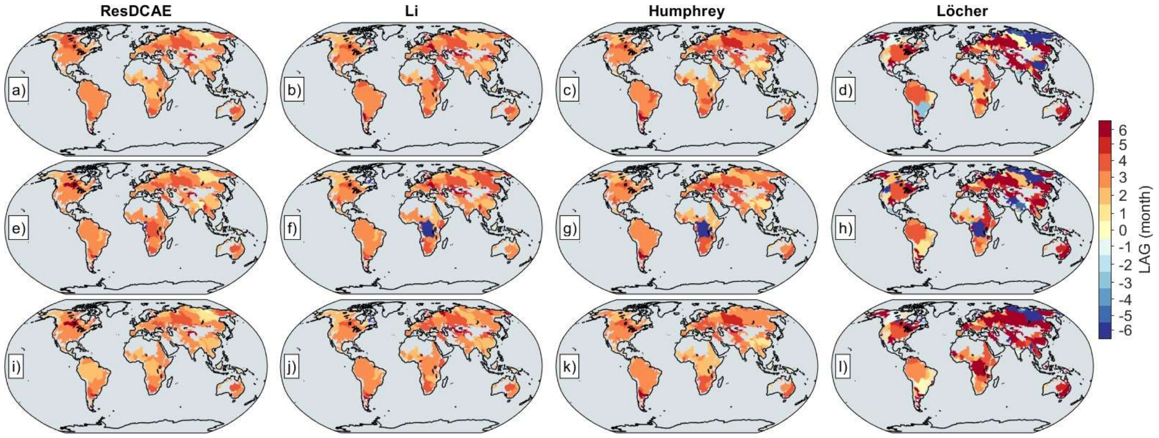

Fig. S6 Time lags of the maximum correlations computed between mass anomaly and precipitation time series presented in Fig. 6.

References

1. Li, F., Kusche, J., Chao, N., Wang, Z. & Löcher, A. Long-term (1979-present) total water storage anomalies over the global land derived by reconstructing GRACE data. *Geophys. Res. Lett.* **48**, e2021GL093492 (2021).

2. Humphrey, V. & Gudmundsson, L. GRACE-REC: a reconstruction of climate-driven water storage changes over the last century. *Earth Syst. Sci. Data* **11**, 1153–1170 (2019).

3. Löcher, A. & Kusche, J. A hybrid approach for recovering high-resolution temporal gravity fields from satellite laser ranging. *J. Geod.* **95**, 6 (2021).

4. Oki, T. & Sud, Y. C. Design of total runoff integrating pathways (trip)—a global river channel network. *Earth Interactions* **2**, 1–37 (1998).
